# Long-term health conditions and UK labour market outcomes during the COVID-19 pandemic

**DOI:** 10.1371/journal.pone.0302746

**Published:** 2024-05-10

**Authors:** Edward J. D. Webb, Philip G. Conaghan, Max Henderson, Claire Hulme, Sarah R. Kingsbury, Theresa Munyombwe, Robert West, Adam Martin

**Affiliations:** 1 Leeds Institute of Health Sciences, Academic Unit of Health Economics, University of Leeds, Leeds, United Kingdom; 2 Leeds Institute of Rheumatic and Musculoskeletal Medicine, University of Leeds, Leeds, United Kingdom; 3 National Institute for Health Research (NIHR) Leeds Biomedical Research Centre, Leeds, United Kingdom; 4 Leeds Institute of Health Sciences, University of Leeds, Leeds, United Kingdom; 5 Department of Health & Community Sciences, University of Exeter Medical School, University of Exeter, Exeter, United Kingdom; 6 Leeds Institute of Cardiovascular and Metabolic Medicine, University of Leeds, Leeds, United Kingdom; 7 Faculty of Health and Social Sciences, Leeds Beckett University, Leeds, United Kingdom; Sungkyunkwan University School of Social Sciences, REPUBLIC OF KOREA

## Abstract

**Background:**

Long-term health conditions can affect labour market outcomes. COVID-19 may have increased labour market inequalities, e.g. due to restricted opportunities for clinically vulnerable people. Evaluating COVID-19’s impact could help target support.

**Aim:**

To quantify the effect of several long-term conditions on UK labour market outcomes during the COVID-19 pandemic and compare them to pre-pandemic outcomes.

**Methods:**

The Understanding Society COVID-19 survey collected responses from around 20,000 UK residents in nine waves from April 2020-September 2021. Participants employed in January/February 2020 with a variety of long-term conditions were matched with people without the condition but with similar baseline characteristics. Models estimated probability of employment, hours worked and earnings. We compared these results with results from a two-year pre-pandemic period. We also modelled probability of furlough and home-working frequency during COVID-19.

**Results:**

Most conditions (asthma, arthritis, emotional/nervous/psychiatric problems, vascular/pulmonary/liver conditions, epilepsy) were associated with reduced employment probability and/or hours worked during COVID-19, but not pre-pandemic. Furlough was more likely for people with pulmonary conditions. People with arthritis and cancer were slower to return to in-person working. Few effects were seen for earnings.

**Conclusion:**

COVID-19 had a disproportionate impact on people with long-term conditions’ labour market outcomes.

## Introduction

The UK Department of Health defines a long-term condition as “a condition that cannot, at present, be cured but is controlled by medication and/or other treatment/therapies” [1]. Large numbers of people in the UK live with a long-term condition, for example it is estimated that 20.3 million people have arthritis [2] and 1 in 6 people have a mental health condition [3], while 15.9% of the population have asthma [4], and 5.2% have diabetes [5]. Living with a long-term health condition can affect labour market outcomes, including reduced working hours and earnings [6–11]. The COVID-19 pandemic, and reaction to it, caused large-scale upheaval to the labour market [12–15], with substantial increases in remote working [16] and 11.7 million people in the UK being placed on furlough [17]. Given this disruption, it is important to revisit previous findings and examine whether the effect of having a long-term condition on labour market outcomes changed after COVID-19’s onset.

There are many ways in which pandemic-related disruption could have impacted people with long-term health conditions more severely. For example, previous research has shown that employers can be reluctant to employ people with long-term conditions due to real or perceived lower productivity [18,19]. In the pandemic this could potentially have led to a greater likelihood of furlough, or even dismissal, for employees with health conditions. People with long-term conditions may also have felt more at risk from COVID-19 [20,21], and felt obliged to withdraw from work to protect themselves. Another reason people with health conditions may have been disproportionately affected is that new ways of working, rapidly introduced, failed to accommodate their needs. It could also be that health and support services, as well as routine care were harder or impossible to access during the pandemic [22–25].

Examining possibilities like the ones above is a prerequisite for targeting labour market policies to better support people with long-term conditions, and to ensure that interventions are as effective as possible in a post-COVID-19 environment. Knowing who, if anyone, was disproportionately affected by the COVID-19 pandemic, and in what ways, is vital in creating a well-functioning post-COVID-19 labour market.

This article addresses the knowledge gap by quantifying the labour market experiences of UK people with several different long-term health conditions both before and during the COVID-19 pandemic using a large longitudinal dataset [26,27]. We studied whether people employed in January/February 2020 with a given long-term condition left employment at a greater rate than people with similar characteristics but without the condition over the first 18 months of the pandemic. We also studied the intensity of participation by examining hours worked conditional on employment. These results were then contrasted with results for a two-year period before the pandemic. COVID-19 changed the ways many people experienced labour market participation, and so we also looked at whether people with long-term conditions were more likely to be furloughed, thus experiencing employment without actively working, or were more likely to work from home. Finally, we studied whether the returns to labour market participation in the form of earnings conditional on employment were lower for people with a long-term condition, both during COVID-19 and over a similar time-span pre-pandemic.

By studying multiple conditions, we were able to get a broad picture of the scale of the challenges faced by people with health conditions. It also allowed us to compare experiences between different populations, and elicit similarities and differences. Comparing pre- and post-COVID-19 outcomes allowed us to examine to what extent the pandemic changed the employment landscape for people with long-term conditions. It also provided evidence as to what extent COVID-19 had a causal impact on outcomes.

While this is the first paper we are aware of to study the labour market outcomes of many different groups of people with long-term conditions during COVID-19, some research has examined UK labour market outcomes during the pandemic for people with disabilities [22,28,29]. While this is a separate population to the ones we study, there is some overlap, and disabled people and people with long-term conditions may have faced similar issues. Our findings are compared and contrasted with previous and ongoing research on disability and the labour market during COVID-19 in the discussion section.

## Methods

All analyses were carried out in R version 4.1.1 [30]. Ethical approval was not necessary for this study, as it used only anonymised data available for download under an end user license [31]. Participant consent was not required for this study, but informed consent to take part in the Understanding Society study was obtained, for details see https://www.understandingsociety.ac.uk/documentation/mainstage/consents/ (accessed 15/3/24).

### Data

The UK Household Longitudinal Survey (UKHLS, also known as Understanding Society) [27] is a longitudinal survey which collects information from individuals living in around 40,000 households on a broad range of topics. It began in 2009 as a successor to the British Household Panel Survey, and there are currently 12 waves of data available. Data collection for a new wave starts each year and lasts for around two years, so that the different waves overlap. Wave 9 was the last to survey participants entirely prior to the COVID-19 pandemic (January 2017 –May 2019), with waves 10 (January 2018 –May 2020) and 11 (January 2019 –May 2022) collecting data both before and after its onset.

The UKHLS COVID-19 survey [26] aimed to collect a more frequent and focused picture of UK society during the pandemic. All UKHLS participants were invited to answer a shorter (approximately 20 minutes) survey, which the majority self-completed online and the remainder completed via telephone interview. There were nine waves in total, with each wave’s data collection lasting a month. The waves were monthly from April 2020-July 2020, bimonthly from September 2020-March 2021, and the final wave was six months later in September 2021. Participants answered a range of questions about their experiences of the pandemic. In the first wave in which they participated, they also gave some information about their lives at baseline, i.e. January/February 2020.

In the main survey individuals were asked questions about their health, including whether they had ever experienced a range of long-term health conditions (“Has a doctor or other health professional ever told you that you have any of these conditions?”). We classed people as having a given condition if they indicated having it in any main survey wave with a response date pre-March 2020. On the advice of clinical co-authors, we grouped together some similar conditions (e.g. different types of arthritis) to provide larger sample sizes. We opted to exclude conditions experienced by fewer than 100 eligible participants, judging this as too few to analyse. We also excluded hyperthyroidism and hypothyroidism, on the advice of clinical co-authors that they were unlikely to have a significant impact on labour market outcomes. Full details about how conditions were grouped and selected are given in S1 Table. This left the following conditions/groups of conditions for analysis:-

1. asthma;
2. arthritis;
3. cancer;
4. diabetes;
5. emotional, nervous or psychiatric problem (ENP);
6. vascular conditions;
7. pulmonary condition;
8. liver conditions;
9. epilepsy.

Conditions were analysed separately as we anticipated that there would be heterogeneity in terms of the labour market impact. Heterogeneity was expected firstly as previous research has shown that people with different long-term conditions’ labour market outcomes were impacted in different ways and to different extents even before the pandemic [18,32–34]. Secondly, COVID-19 had potential to affect people with different conditions in many different ways. For example, people with conditions that affect their immune or respiratory systems may have felt especially vulnerable, leading to a different impact compared to people with conditions that did not imply an increased vulnerability to COVID-19. However, given the unprecedented disruption to work that COVID-19 caused, we did not have prior expectations as to who would be affected or in what way.

### Analysis

The aim of the analysis was to investigate the causal effect of having a given long-term condition on labour market outcomes during the COVID-19 pandemic. To control for differences in observable characteristics between people with and without a given long-term condition, we used a doubly robust approach [35]. This involved first using matching to create samples which had similar baseline characteristics. Second, to control for any remaining differences, we included individual characteristics as covariates in regression analyses. To control for differences in unobserved characteristics, a random effects parameter was included in all longitudinal analyses.

To examine the effect of COVID-19, it is necessary to compare outcomes to what would have happened in the absence of the pandemic. It is more difficult to create a formal counterfactual in this case. However, to provide context to pandemic-era results, we also conducted a pre-COVID-19 analysis using waves 7–9 of the main survey, the last three waves which took place entirely before 2020. This analysis studies how people with long-term conditions’ labour market outcomes evolved over a two-year period, comparable to the 18 months covered by the COVID-19 survey. We argue that if larger effects are seen in the COVID-19 results than in the pre-COVID-19 analysis, then this is likely attributable to the pandemic.

### Mahalanobis distance matching

In the COVID-19 analysis, we included participants who, in the COVID-19 survey, indicated being in employment (including self-employment) at baseline (January/February 2020). For the pre-COVID-19 analysis we included participants who were employed in wave 7 of the main survey. No exclusions were made based on age, as many survey participants who had reached the typical age of retirement were still participating in the labour market: around 12.5% of participants over 65 reported being in employment in at least one survey wave.

We created samples of people with and without given conditions matched on baseline variables using nearest neighbour Mahalanobis distance matching (MDM) without replacement as implemented in the MatchIt package for R [36]. MDM matches treatment/control observations based on measuring the Mahalanobis distance between them, a measure of how similar they are on all variables weighted by the amount of variation in each variable. MDM is similar to the commonly used method of propensity score matching (PSM), which estimates how likely observations are to be in the treatment group and matches treatment/control observations with similar probabilities. However, MDM is more likely to create samples which are balanced on all variables, whereas PSM may result in matched individuals with similar propensity scores, but with wide differences on some variables [37].

Details about what variables were matched on are given in Table 1. Variables for matching were selected based on consulting previous literature [e.g. 38,39], as well as consideration of what characteristics, such as key-worker status, would be relevant during the pandemic. We also matched based on baseline outcome variables. The variables were similar in the COVID-19 and pre-COVID-19 analysis, however, the latter excluded baseline working from home and key-worker status due to these variables not being collected. It also excluded baseline universal credit due to the incomplete rollout of the universal credit programme when data collection took place.

**Table 1:** Independent and matching variables.

Variable	Definition	Survey waves		Time varying?
		COVID-19 analysis	Pre-COVID-19 analysis	
Age^a^	Age in years	C1-9	M7-9	Y
Sex	1 if female, 0 otherwise	C1-9	M7-9	N
Ethnicity	1 if white, 0 otherwise	C1-9	M7-9	N
Baseline hours worked	Number of hours usually worked (including self-employed) in January/February 2020 (COVID-19 analysis)/main survey wave 7 (pre-COVID-19 analysis)	C1-5^b^	M7	N
Baseline earnings	Annualised take-home pay (including self-employed) in £1,000s in January/February 2020 (COVID-19 analysis)/main survey wave 7 (pre-COVID-19 analysis)	C1-5^b^	M7	N
Baseline household earnings	Annualised take-home household pay in £1,000s in January/February 2020 (COVID-19 analysis)/main survey wave 7 (pre-COVID-19 analysis)	C1-5^b^	M7	N
Baseline work from home	How often worked from home in January/February 2020. 1 = always, 2 = often, 3 = sometimes, 4 = never; re-coded as 1 = always, 2 = hybrid, 3 = never for analysis	C1-5^b^	-	N
Baseline universal credit	1 if receiving universal credit in January/February 2020, 0 otherwise	C1-5^b^	-	N
Key-worker	Whether self-identify as key-worker	C1-2^c^	-	N
Location	NUTS level 1 region	C1-9	M7	Y
Household size^d^	Number of people in household	C2-9	M7-9	Y
Job type^e^	Three category NS-SEC job type (management and professional, intermediate, routine)	M10	M7-9	Y
Baseline number of comorbidities	Number of long-term conditions participants reported having, excluding condition being analysed	M1-11	M1-6	N

*Note*. C indicates COVID-19 survey waves, M indicates main survey waves; N = no; Y = yes; MDM = Mahalanobis distance matching; NUTS = Nomenclature of Territorial Units for Statistics; NS-SEC = National Statistics Socio-economic Classification.

^a^ Log of age used in regression models.

^b^ Only asked if no response in previous waves.

^c^ Online only wave 1, phone only wave 2.

^d^ Household size was not collected in COVID-19 survey wave 1, so it was assumed to be the same as in wave 2.

^e^ Job type was not collected in COVID-19 survey, so for COVID-19 analysis, baseline job type collected in main survey wave 10 was used.

Where there were few people with a condition, it was possible to find several people without it who made suitable controls, but this became more difficult when more people had the condition. Therefore, the matching ratio depended on the number of people with the condition. For 150 or fewer participants, the ratio was 4:1 (i.e. four controls matched to every participant with the condition), with 150–500 participants, the ratio was 3:1, for 500–1,000 participants, it was 2:1, and for more than 1,000 participants it was 1:1.

The baseline matching procedure required complete information on all baseline variables. Rather than discard all responses from respondents missing one or more baseline variables, missing baseline variables were replaced using random forest multiple imputation, as implemented in the MissForest package [40] with 100 trees and a maximum of 100 iterations.

### Modelling

Outcomes were studied separately for each long-term condition and for the COVID-19 and pre-COVID-19 periods. The outcomes were selected to give a broad overview of people’s experience of the labour market during COVID-19: participation, furlough, working patterns, and returns to participation. We did not have any firm expectation of which groups might be affected, or how, given the unprecedented disruption of the pandemic. Nor did we have an expectation that people with long-term conditions would necessarily be worse off, given for example the potential opportunities afforded by increased working from home.

To assess the impact of having a long-term condition on labour market participation, we chose employment status (including self-employed), as a dependent variable, as well as number of hours worked conditional on employment, in line with previous research [38]. Both of these outcomes were examined using random-effects models, with equations of the form

$$y_{it}=\beta_{0}+\beta_{t}t+\beta_{LTC}{LTC}_{i}+\beta_{LTC\times t}{LTC}_{i}\times t+\beta_{X}X_{it}+\beta_{Z}Z_{i}+\nu_{i}+\varepsilon_{it}$$ (1)

where *y*_*it*_ is the outcome for individual *i* at time *t*, *LTC*_*i*_ is a dummy variable equal to 1 if *i* has a given long-term condition and 0 otherwise, ***X***_***it***_ and ***Z***_***i***_ are vectors of respectively time varying and time invariant control variables, $\nu_{i}∼N(0,\sigma_{\nu }^{2})$ is a normally distributed individual effect, *ε*_*it*_ is an error term and the *β*s are coefficients to be estimated. The time variable *t* was defined differently in the COVID-19 and pre-COVID-19 analyses. In the COVID-19 analysis, *t* was defined as months after April 2020, i.e. *t* = 0 is the first wave of the Understanding Society COVID-19 survey. In the pre-COVID-19 analysis, *t* = 0,1,2 corresponds to waves 7, 8 and 9 respectively of the main Understanding Society survey. Control variables were the same as the matching variables in Table 1. These controls were included as an additional way of ensuing balance between people with/without a given long-term condition. As the goal was to create comparable samples of people with/without a given long-term condition to increase the likelihood of identifying causal effects, rather than a representative sample of the UK population, UKHLS sample weights were not used. Logit models were used for employment and Tobit models were used for hours worked, which are standard ways of analysing respectively binary and censored dependent variables [41].

People who were furloughed during the pandemic experienced labour market participation in a fundamentally different way, remaining employed but not actually working. To see if people with a long-term condition were more likely to belong to this group, we used whether participants were furloughed at any point as a dependent variable in cross-sectional logit models of the form

$$y_{i}=\beta_{0}+\beta_{LTC}{LTC}_{i}+\beta_{X}X_{i}+\varepsilon_{i}.$$

Another way in which experience of labour market participants changed during the pandemic was increase in working from home, so frequency of working from home was also analysed using ordered logit models, a standard approach for categorical outcomes with a natural ordering [42], with the same functional form as Eq (1). (Furlough and working from home were not examined in the pre-COVID-19 analysis due to the former not existing and no data on the latter).

Finally, to see if returns to labour market participation for people with long-term conditions were disproportionately affected earnings conditional on employment was studied using Tobit models with the same functional form as Eq (1).

Full details as to how the dependent variables were defined and how they were analysed are given in Table 2. Coefficients were assessed using *t*-tests with statistical significance judged at the conventional 5% level.

**Table 2:** Dependent variables.

Variable	Definition	Survey waves		Model
		COVID-19 analysis	Pre-COVID-19 analysis	
Employed	1 if employed (including self-employed), 0 otherwise	C1-9	M7-9	RE panel logit^c^
Hours worked	Number of hours worked (including self-employed) in previous week conditional on employment	C1-9	M7-9	RE panel Tobit^d^
Furlough	Whether individual reports being furloughed in any COVID-19 survey wave	C1-8^a^	N/A	CS logit^f^
Working from home	How often worked from home during the previous four weeks. 1 = always, 2 = often, 3 = sometimes, 4 = never; re-coded as 1 = always, 2 = hybrid, 3 = never for analysis	C1-9	N/A	RE panel ordered logit^e^
Earnings	Annualised take-home pay (including self-employed) in £1,000s conditional on employment	C1-9	M7-9	RE panel Tobit^d^

*Note*. RE = random effects; LPM = linear probability model; CS = cross-sectional; C indicates COVID-19 survey waves, M indicates main survey waves.

^a^Combination of several variables, see “Constructing a furlough related variable” in UKHLS COVID-19 survey FAQs, https://www.understandingsociety.ac.uk/documentation/covid-19/faqs.

^b^Only asked if never reported receiving universal credit in previous waves.

^c^Estimated using the pglm package [30].

^d^Estimated using the censReg package [31].

^e^Estimated using Apollo Choice Modelling package [32].

^f^Estimated using the glm command from the stats package.

## Results

Table 3 provides descriptive baseline statistics for the COVID-19 analysis samples before and after imputing missing baseline variables, and Table 4 provides them for the pre-COVID-19 analysis. A total of 2,867 responses (4.73%) in the pre-COVID-19 sample were obtained by proxy interviews; proxy responses were not possible in the COVID-19 survey. For personal characteristics in the COVID-19 data and all variables in the pre-COVID-19 data there were few missing responses. For job and financial related responses in the COVID-19 data, there were greater fractions of missing observations, for example 18.8% of participants had a missing job type, and 9.4% had missing information about earnings.

**Table 3:** Summary of COVID-19 data.

		Pre-imputation mean	Fraction missing	Post-imputation mean
Age (years)		45.2	0%	45.2
Female		57.7%	0.1%	57.7%
Ethnicity—White		85.8%	1.2%	85.8%
Hours worked		33.4	0.7%	33.4
Earnings (£1,000s)		22.3	9.4%	22.2
Work from home	always	6.5%	1.1%	6.5%
	hybrid	26.3%		26.5%
	never	67.1%		67%
Job type	professional and managerial	49.8%	18.8%	47.7%
	intermediate	23.4%		23.2%
	routine	26.9%		29.1%
Universal credit		1.9%	5.9%	1.8%
Key worker		41.8%	12.2%	42.4%
Location	North East	3.3%	0.0%	3.3%
	North West	9.5%		9.5%
	Yorkshire	8.5%		8.55
	East Midlands	7.5%		7.5%
	West Midlands	8.3%		8.3%
	East England	9.5%		9.5%
	London	12.1%		12.1%
	South East	13.4%		13.4%
	South West	8.9%		8.9%
	Wales	5.8%		5.8%
	Scotland	8.7%		8.7%
	Northern Ireland	4.6%		4.6%
Household size		3	22.4%	3
Household income (£1,000s)		39.8	15.9%	39.6

*Note*. *N* = 12,432.

**Table 4:** Summary of pre-COVID-19 data.

		Pre-imputation mean	Fraction missing	Post-imputation mean
Age (years)		43	0%	43
Female		49.8%	0%	(49.8)
White		80%	0.4%	(80.9)
Hours worked		37.1	0%	37.1
Earnings (£1,000s)		18.8	0%	18.8
Job type	professional and managerial	41.3%	1.1%	42.0%
	intermediate	23.4%		24.8%
	routine	32.5%		33.2%
Location	North East	3.3%	0%	3.3%
	North West	9.8%		9.8%
	Yorkshire	8.3%		8.3%
	East Midlands	7.1%		7.1%
	West Midlands	8.2%		8.2%
	East England	8.3%		8.3%
	London	14.8%		14.8%
	South East	12.2%		12.2%
	South West	7.8%		7.8%
	Wales	6.1%		6.1%
	Scotland	8.2%		8.2%
	Northern Ireland	5.8%		5.8%
Household size		3.2	0.3%	3.2
Household income (£1,000s)		48.6	0.3%	48.6

*Note*. *N* = 23,388.

S2–S10 Tables and S16–S24 Tables give detailed results about the MDM procedure for all conditions for respectively the COVID-19 and pre-COVID-19 analysis samples. In general, control and treatment samples were well matched, with the greatest standardised mean differences (SMDs) seen for number of comorbidities. The SMD for this variable ranged between 0.0614 for cancer and 0.837 for asthma, both in the COVID-19 analysis samples. Table 5 shows how many people were included in the treatment and control samples for each condition. The most prevalent condition was asthma, with over 5,000 respondents reporting the condition, and the least common, with only 119 people, was pulmonary conditions.

**Table 5:** Number of people in treatment and matched control groups for each condition.

Condition	COVID-19 analysis			Pre-COVID-19 analysis		
	Treatment	Control	Total	Treatment	Control	Total
Asthma	5104	5104	10208	9010	9010	18020
Arthritis	2304	2304	4608	4222	4222	8444
Cancer	553	1106	1659	1033	1033	2066
Diabetes	604	1208	1812	1327	1327	2654
ENP	1517	1517	3034	2271	2271	4542
Vascular	2241	2241	4482	4426	4426	8852
Pulmonary	119	597	716	434	1302	1736
Liver	251	753	1004	496	1488	1984
Epilepsy	130	520	650	245	735	980

Table 6 summarises model results, with full results given in S11–S15 Tables and S251–S27 Tables. For time-varying dependent variables, the long-term condition coefficient in the COVID-19 analysis represents the initial impact (if any) on people with long-term conditions’ outcomes compared to a matched control group in the immediate aftermath of the pandemic’s onset in the UK, i.e. April 2020. It thus captures whether COVID-19 initially affected everyone in the same way, or whether people with a given long-term condition were affected differently. In the pre-COVID-19 analysis, the coefficient shows the divergence between people with long-term conditions’ outcomes compared to the matched control group between baseline (main survey wave 7) and time 0 (main survey wave 8). The interaction between the long-term condition variable and time then shows the amount the initial effect changed each month (COVID-19 analysis) or wave (pre-COVID-19 analysis). In the COVID-19 analysis, there were significant and negative initial effects of ENP and liver conditions on probability of employment, and for each the time-trend interactions were small and statistically insignificant. This indicates that the initial reduction in employment rates for people with those conditions persisted throughout the pandemic period. For asthma, arthritis, vascular conditions, and epilepsy there were statistically significant and negative interactions of condition with time trend. This indicates that the gap between the probabilities of people with and without a condition being employed grew during the pandemic. No long-term condition coefficients or time-trend interactions were significant in the pre-COVID-19 models. Hence no significant divergence in employment rates between people with and without long-term conditions over that two-year pre-pandemic period was observed.

**Table 6:** Results summary.

	Asthma		Arthritis		Cancer		Diabetes		ENP		Vascular		Pulmonary		Liver		Epilepsy	
	Coeff.	*p*	Coeff.	*p*	Coeff.	*p*	Coeff.	*p*	Coeff.	*p*	Coeff.	*p*	Coeff.	*p*	Coeff.	*p*	Coeff.	*p*
Probability of employment																		
COVID-19 analysis																		
LTC	-0.143	0.415	-0.0338	0.876	-0.437	0.18	0.717	0.088	-0.648	0.017*	0.195	0.406	0.394	0.408	-1.07	0.010*	-0.473	0.474
*t*	-0.0952	0.000*	-0.135	0.000*	-0.172	0.000*	-0.16	0.000*	-0.114	0.000*	-0.142	0.000*	-0.185	0.000*	-0.106	0.000*	-0.0545	0.015*
LTC × *t*	-0.0406	0.000*	-0.032	0.019*	-0.0278	0.231	-0.0427	0.075	0.0111	0.513	-0.0359	0.013*	-0.0157	0.71	9.61x10^-3^	0.777	-0.19	0.000*
Pre-COVID-19 analysis																		
LTC	0.232	0.449	0.192	0.668	-0.328	0.716	-0.799	>.999	-0.417	0.474	-0.298	0.504	0.736	>.999	-0.712	>.999	0.566	>.999
*t*	-1.2	0.000*	-1.27	0.000*	-1.43	0.002*	-1.29	>.999	-1.17	0.000*	-1.41	0.000*	-1.01	>.999	-1.03	>.999	-1.4	>.999
LTC × *t*	-0.0851	0.667	-0.0627	0.826	0.164	0.779	0.0513	>.999	0.0376	0.918	0.118	0.685	-0.294	>.999	0.0282	>.999	-0.219	>.999
Hours worked conditional on employment																		
COVID-19 analysis																		
LTC	-0.0463	0.847	0.182	0.587	-1.02	0.065	-1.13	0.029*	-0.497	0.218	-0.406	0.235	-2.46	0.023*	-0.161	0.839	-1.39	0.251
*t*	0.559	0.000*	0.594	0.000*	0.502	0.000*	0.566	0.000*	0.576	0.000*	0.534	0.000*	0.63	0.000*	0.652	0.000*	0.535	0.000*
LTC × *t*	7.43x10^-3^	0.647	-0.0589	0.017*	0.0672	0.086	0.0318	0.421	-0.0283	0.332	0.0145	0.57	-0.0622	0.408	-0.11	0.07	0.11	0.206
Pre-COVID-19 analysis																		
LTC	0.194	0.535	0.0416	0.93	0.352	0.672	-0.0854	0.925	-0.253	0.713	0.087	0.846	-0.0712	0.958	-0.379	0.739	-0.22	0.894
*t*	0.275	0.032*	0.318	0.095	-0.492	0.162	0.349	0.378	0.803	0.002*	-0.0141	0.94	0.226	0.544	0.65	0.041*	0.516	0.269
LTC × *t*	0.048	0.782	7.59x10^-3^	0.977	-0.0444	0.925	-0.268	0.597	-0.244	0.516	0.179	0.471	-0.0834	0.906	-0.391	0.534	0.273	0.755
Probability of furlough																		
COVID-19 analysis																		
LTC	-0.0309	0.596	5.98x10^-3^	0.939	0.152	0.267	0.0475	0.716	-0.13	0.168	0.0792	0.316	0.542	0.006*	0.254	0.184	0.199	0.427
Working from home frequency																		
COVID-19 analysis																		
LTC	-0.0105	0.913	-0.209	0.083	0.0986	0.62	0.0226	0.912	0.145	0.275	-4.58x10^-4^	0.997	0.216	0.509	-0.0952	0.734	0.139	0.722
*t*	-0.0581	0.000*	-0.0624	0.000*	-0.0592	0.000*	-0.0573	0.000*	-0.0588	0.000*	-0.0596	0.000*	-0.0583	0.000*	-0.0582	0.000*	-0.0577	0.000*
LTC × *t*	6.32x10^-4^	0.917	0.0251	0.001*	0.031	0.042*	-0.0133	0.34	7.58x10^-3^	0.419	9.47x10^-3^	0.213	0.035	0.173	0.0158	0.453	-0.0143	0.661
Earnings conditional on employment																		
COVID-19 analysis																		
LTC	-0.224	0.241	-0.244	0.457	0.265	0.561	-1.16	0.105	-0.744	0.067	0.238	0.496	0.376	0.756	-0.696	0.434	-1.64	0.119
*t*	0.124	0.000*	0.0799	0.000*	0.0961	0.000*	0.0859	0.013*	0.0727	0.021*	0.133	0.000*	0.131	0.035*	0.223	0.000*	0.115	0.020*
LTC × *t*	-0.0204	0.255	0.0612	0.018*	0.0709	0.040*	0.0491	0.433	0.067	0.084	-0.0155	0.577	-0.0652	0.523	-0.0172	0.85	-0.0595	0.437
Pre-COVID-19 analysis																		
LTC	0.0412	0.834	-0.0285	0.914	0.17	0.798	-0.0225	0.966	-4.26x10^-3^	0.991	0.0338	>.999	0.0269	0.967	-0.28	0.717	0.0558	0.952
*t*	0.58	0.000*	0.487	0.000*	0.165	0.526	0.516	0.018*	0.715	0.000*	0.48	>.999	0.409	0.029*	0.529	0.008*	0.647	0.027*
LTC × *t*	-0.195	0.061	-0.16	0.26	0.0972	0.788	-0.446	0.12	-0.0812	0.691	-0.111	>.999	-0.129	0.717	0.012	0.977	0.014	0.979

*Note*. LTC = Long-term condition; in COVID-19 analysis, *t* = months after April 2020, in pre-COVD-19 analysis, *t* = 0,1,2 signifies Understanding Society main survey waves 7, 8, 9; ENP = emotional, nervous or psychiatric problem; coeff. = coefficient; covariates listed in Table 1; full results in S11–S15 Tables (COVID-19 analysis) and S25–S27 Tables (pre-COVID-19 analysis).

For all participants in the COVID-19 analysis there was a reduction in hours worked conditional on employment in April 2020 compared to January/February 2020. Table 6 shows that there was a significantly negative time-trend interaction for arthritis, indicating that people with arthritis were slower than matched controls to increase their working hours following the initial drop. The significant coefficients for diabetes and pulmonary conditions shows that the initial reduction in hours was greater for participants with either condition compared to matched controls. For each, the time-trend interaction was also small and statistically insignificant, indicating they continued to work fewer hours compared to matched controls throughout the pandemic. Table 6 also summarises the pre-COVID-19 analysis for hours worked conditional on employment. There are no significant long-term condition coefficients of time-trend interactions.

**Fig 1:**
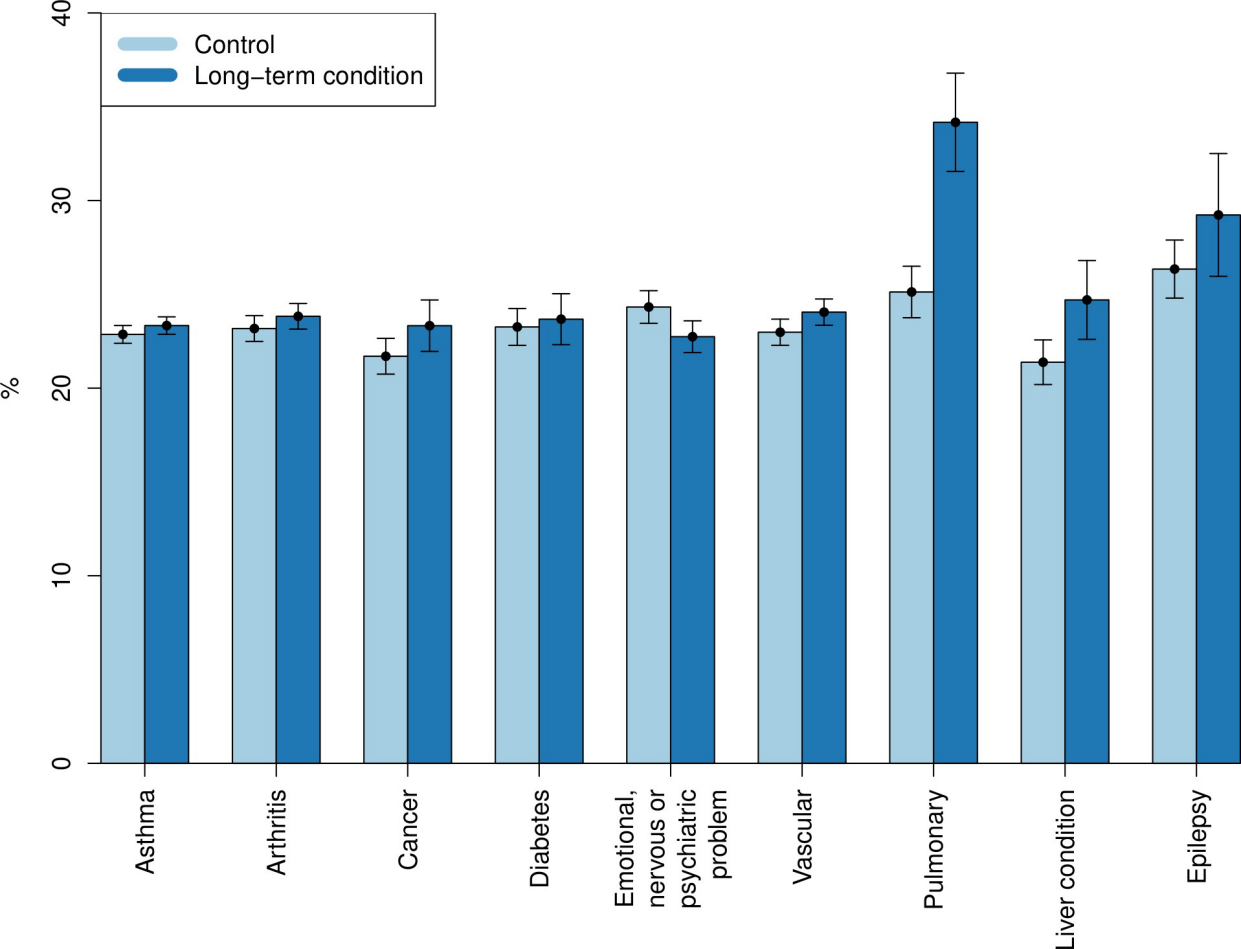
Fraction of respondents who were ever furloughed.

Fig 1 shows the differences between how likely people with a long-term condition and matched controls were to be furloughed at some point in the pandemic. The greatest difference is seen for participants with pulmonary conditions. This observation is confirmed by Table 6, where the only significant effect in the analysis of probability of being furloughed is found for that patient group. The odds of being furloughed were 1.7 greater for people with pulmonary conditions compared to matched controls.

**Fig 2:**
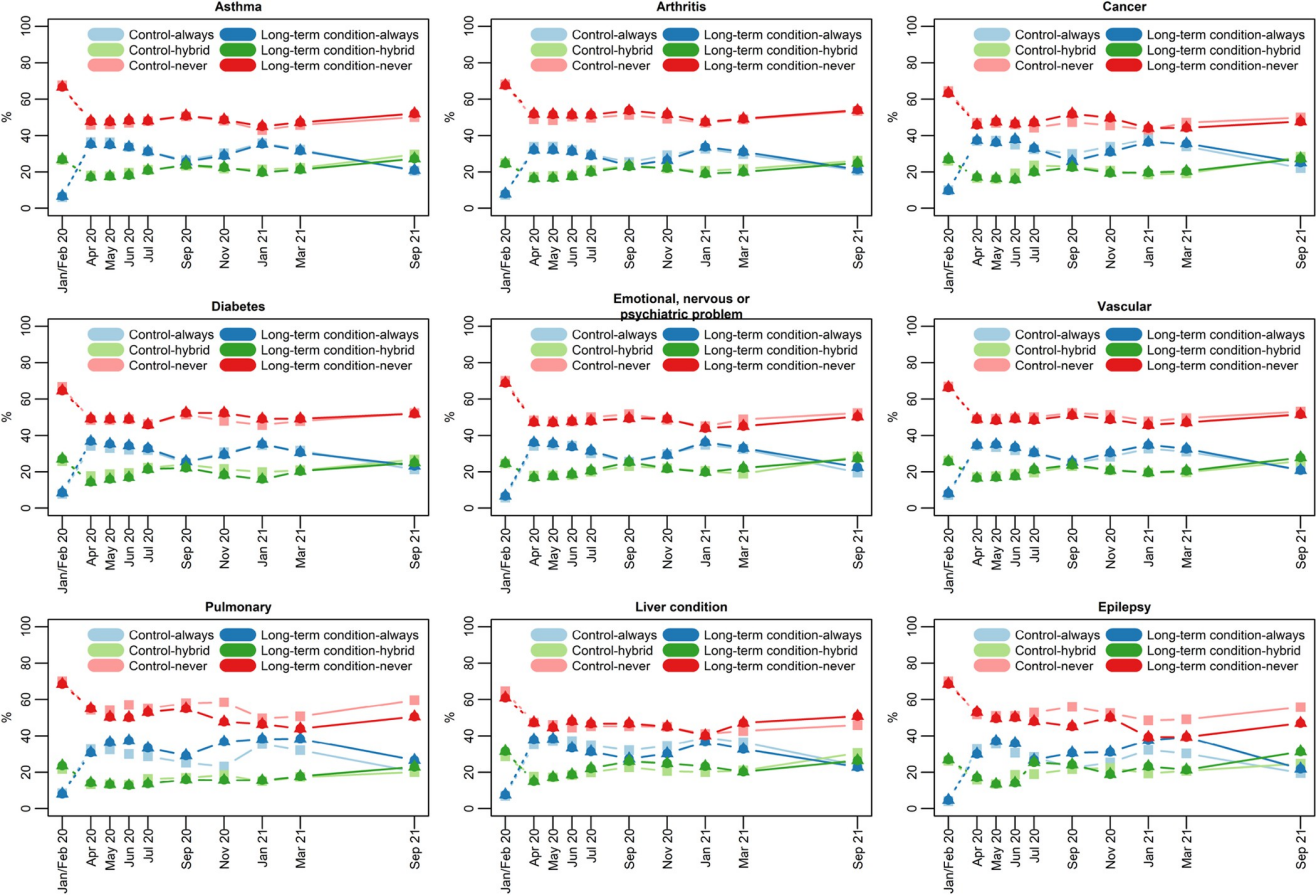
Working from home frequency.

Fig 2 illustrates where participants in the COVID-19 analysis worked, conditional on employment: always at home, hybrid, or never from home. There was a large rise in participants always working from home in April 2020 compared to baseline, and a corresponding fall in people who never worked from home. This pattern was still notably different from baseline in the final survey wave. Table 6 shows that there was no significant initial effect on working from home frequency for any long-term condition. However, for two conditions, arthritis and cancer, there were positive and significant time-trend interactions, showing they were slower to return to in-person working compared to matched controls.

Table 6 shows that at the onset of COVID-19 there were no significant differences between the earnings conditional on employment of people with any studied conditions and their matched controls. For two conditions (arthritis and cancer), there was a significant and positive interaction with the time trend, though note that in each case the sign of the initial effect is negative. This could represent a recovery following an initial drop in earnings, which the analysis was not sufficiently powered to detect at the chosen significance level. In the pre-COVID-19 analysis, there were no significant effects of having a long-term condition on earnings conditional on employment.

## Discussion

The results illustrate substantial disruption to the UK labour market since March 2020. While the broad patterns were the same for people with and without long-term conditions, there were indications that people with long-term conditions were disproportionately affected during the 18 months following the pandemic’s outbreak.

The most common way in which people with long-term conditions were affected was that they were more likely to leave employment. Previous research has shown that many conditions presented barriers to work prior to the pandemic [38,43–46], so it may appear as if the decline in employment rates was unrelated to COVID-19. However, we studied only people with long-term conditions in employment at baseline, and compared their outcomes to people with similar characteristics. We also studied a relatively short space of time in the COVID-19 analysis, so previous research does not necessarily imply that a significant employment gap should have opened up regardless of the pandemic occurring. Indeed, in the pre-COVID-19 analysis, no significant employment gap between people with long-term conditions, and matched controls was seen over a comparable period. We then interpret the decline in the COVID-19 analysis as a causal effect of the pandemic: COVID-19 has exacerbated work barriers for people with long-term conditions.

Previous research has suggested that unemployment can have a causal and negative impact on people’s health, particularly mental health [47–50]. For people with long-term conditions who left employment during COVID-19, there is hence a risk of further ill health. This in turn may further reduce their chances of re-entering the labour market.

For some conditions, the intensity of labour market participation was also affected, with those in employment working fewer hours than matched controls during the pandemic. Again, no analogous results were seen in the pre-COVID-19 analysis was seen, suggesting a causal effect of the pandemic. The largest effect was seen for participants with pulmonary conditions, and those participants were also more likely to be furloughed. Perhaps this is because people with pulmonary conditions were regarded as more at risk from COVID-19 by employers, even compared to people with other long-term conditions. Then where there was flexibility in which employees were furloughed, employees with pulmonary conditions were prioritised for selection.

It could also be that people with pulmonary conditions were among the most cautious during the pandemic, and therefore the most reluctant to return to in-person work. That latter hypothesis is supported by the fact that the pulmonary group likely included the largest proportion of individuals advised to shield (i.e. minimise all physical interactions). All people with severe respiratory conditions were included in this guidance [51]. On the other hand, only people with specific cancers undergoing certain treatments were advised to shield, so the cancer group probably included many people who fell outside the guidance. Likewise, for the vascular group, only women with heart disease who were pregnant were recommended to shield. Other people included in the guidance were those with rare metabolic disorders, on immunosuppression therapies, or who had previously received a whole organ transplant. It seems unlikely these are a large group in our sample. The finding that for all long-term conditions but one there was no increased probability of furlough is comparable to previous findings that people with disabilities were no more likely to be furloughed when controlling for personal and job characteristics [52].

Some people with long-term conditions may have benefitted from increased working from home, although they could also have been negatively impacted. For example, commuting to work might be challenging, both physically and mentally, for individuals with some conditions [53,54]. On the other hand, people with other conditions may have greater need of a specially adapted workspace not available at home. We found little evidence that people with long-term conditions initially opted for different working conditions than their matched counterparts, either by working from home for a greater or lesser extent. This is probably due to initial changes being legally mandated by job type: If a job could be done from home, it had to be, and non-essential jobs which couldn’t be done from home were not done at all. As restrictions were relaxed, the general trend was for increased in-person working, but people with arthritis and cancer did so at a slower rate than matched controls. For the former group, it could be due to them feeling a benefit from avoiding commuting. For both groups, it could be that some people were shielding due to taking immunosuppressant medication, and thus were avoiding in-person work. If that were the case, then an even larger impact would be expected for pulmonary conditions. The time-trend interaction for pulmonary conditions was statistically insignificant, though this may be due to the analysis being underpowered as the coefficient’s magnitude was the largest out of all conditions. Going forward there are both challenges and opportunities in finding new ways of working, and it is necessary to account for the needs and preferences of people with long-term conditions in doing this.

For people with long-term conditions in employment, there was little evidence that an earnings gap developed between them and matched controls, either in the COVID-19 or pre-COVID-19 analysis. Indeed, for some conditions, participants’ earnings increased significantly faster during the pandemic than matched controls, although it is notable that in each case there was a sizeable, albeit statistically insignificant initial drop, so that the higher trend likely represents recouping initial losses. However, the COVID-19 data spanned only 18 months, whereas the effects of the pandemic on promotions, early retirement, etc., may not be seen for some time.

Rising labour market inactivity, especially in the older population is an ongoing policy discussion in the UK [55], with crucial implications for social care costs, growth and prosperity [56–59]. Previous research has shown that ill-health is a contributory factor in increased levels of labour market inactivity [60], via mechanisms of more people being in ill-health [61], and via long COVID-19 affecting employment [62]. Our study complements these findings by identifying another mechanism: people already living with many long-term conditions at the pandemic’s onset have transitioned to labour market inactivity at a greater rate than people without the condition. This study’s contribution to the debate is to highlight the disproportionate impact of the COVID-19 pandemic on employment for people with existing long-term conditions, and the economic benefit that reversing the trend could bring.

There are similarities between the labour market outcomes during COVID-19 for several of the studied groups. However, there are also many differences, for example reduced working hours were seen for participants with only three out of nine studied conditions, and only those with pulmonary conditions were more likely to be furloughed. It is therefore unlikely that a “one fits all” approach to labour market interventions would be particularly effective. Successful interventions should be targeted at the different needs and challenges faced by individuals with different long-term health conditions in order to maximise the individual and economic benefit.

This study is the first of which we are aware to study the impact of long-term conditions, rather than disability, on labour market outcomes during COVID-19. Having a long-term condition is not the same as having a disability, although many long-term conditions create a risk of disability, especially when combined with comorbidities [63,64]. The current study’s findings complement previous research on disability: it includes many people whose long-term condition was successfully managed to the extent of not considering themselves disabled. However, COVID-19 may have had a disproportionate impact on their labour market outcomes compared to people without a long-term condition by disrupting the management of their condition, for example due to changing working conditions, restricted access to support, or the impact of the virus itself. An advantage of studying conditions separately is the potential variation both in the extent to which the condition was manageable prior to the pandemic and the extent to which the pandemic disrupted management strategies.

Comparing our findings for people with long-term conditions with related research on people with disabilities, Emerson et al. [28] found that people with a disability were more likely to work fewer hours and more likely to experience financial stress using the first three waves of UKHLS COVID-19 survey data. Byrne, Barber & Lim [22] found a post-pandemic onset fall in employment rates for people with a mental health-related disability, and here a similar fall was observed for people with an emotional, nervous or psychiatric problem. However, in that paper, the gap between mental health-disabled and non-disabled people had begun to close by the end of 2021, whereas no comparable trend was observed here. Jones [29] found that people with a disability were more likely to be away from work, including being on furlough. Here, only people with pulmonary conditions were significantly more likely to be furloughed, possibly indicating that people who would consider themselves disabled made up a relatively small fraction of other studied conditions. Jones found that the disability pay gap did not increase during the pandemic, just as we found that people with long-term conditions did not experience lower earnings than matched controls. However, the aforementioned paper also reported no increase in the disability employment gap, whereas we found lower probability of employment for people with several long-term conditions. The contrasts above demonstrate that care should be taken in future research to study each group separately, as well as clearly distinguishing between long-term conditions and disability.

Some previous studies using the Understanding Society COVID-19 survey have used a more complex time structure than the linear trend used here, with one coefficient per survey wave (e.g. [39,65]). While that may have led to a more detailed picture given varying restrictions and disease prevalence over 2020–21, it would also have implied many more model coefficients, which may have caused problems with long-term conditions where the sample size was small. In addition, simplifying models’ time structure into an initial impact, then a broad trend over the next 17 months facilitated comparing patterns of results over several outcomes for many long-term conditions. It would be useful for future research, when examining the labour market outcomes during COVID-19 for a specific condition to model time in a more flexible way, and could also link outcomes to fluctuating restrictions and case levels.

It would have been possible to match participants on a greater number of observed variables, for example on details of what industry people worked in, rather than three categories of job type. However, we wished to avoid overfitting [66], and so relied on for example three-category job type combined with key-worker status (thereby effectively giving six categories) to capture the major differences in job situations. As we also wished to include the matching variables as model covariates as an extra step to ensure balance between treatment/control, having fewer matching variables had the advantage of fewer model coefficients in cases where sample sizes were small.

### Strengths/Limitations

This paper has several strengths. We examined a range of long-term health conditions. This has allowed us to explore both similarities and differences in the labour market outcomes during COVID-19 of diverse patient groups. We also used all UKHLS COVID-19 survey waves and studied a range of outcomes to get a broad picture of participants’ work experiences during the pandemic. Several different measures were used to enhance the likelihood of identifying a causal effect of having a long-term condition.

Participants were matched on baseline variables to created similar samples prior to the pandemic, and as a further measure these variables were used as controls in regression analysis. The control variables included many important factors which would be expected to influence labour market outcomes, such as age, gender, and number of co-morbidities. Unobserved differences between participants which were constant over time were controlled for using random effects panel models.

In the COVID-19 survey, some variables had a relatively large faction of missing data. The reasons for this vary, for example with job type the information was taken from wave 10 of the main survey, where not everyone will have participated, at least not before the cut-off point of 28/2/20. Other variables such as key worker status were only asked in a small number of waves. It is a strength of this study that advanced imputation methods were used to replace these missing observations.

It is also a strength of our paper that we study both findings during COVID-19 as well as studying a similar length of time before the pandemic in the pre-COVID-19 analysis. We showed that pre-pandemic, people with long-term conditions who were in employment had similar outcomes to matched controls over a two-year period. This gives greater confidence that any adverse effects on people with long-term conditions’ labour market outcomes in the COVID-19 analysis were due to the pandemic, and not other factors.

Our study also has some weaknesses. The primary weakness is that the observed effects of having a long-term condition could only be identified as causal under the assumption that the analysis strategy eliminated all unobserved differences between groups with and without a given long-term condition. While matching creates balanced samples with respect to the included observables, there may well have been unobserved differences which influenced labour market outcomes. Likewise, random effects models control for unobserved individual characteristics, but only those which are time-invariant and uncorrelated with the independent variables [67]. In addition, we do not provide a formal test of whether COVID-19 had a causal effect on outcomes, since this was not possible with the available data. Instead, pre-COVID-19 results were presented as an indication that the observed divergences between people with/without a given long-term condition were unlikely to have occurred over 18 months in the absence of COVID-19. However, being unable to definitively conclude that the effects of independent on dependent variables are causal is common for observational studies.

There were also limitations in identifying people with different long-term conditions. Some of the studied groups had broad definitions, with potentially large heterogeneity in people’s experience of their condition. For example, emotional, nervous, or psychiatric problems encompasses many diverse mental health conditions, including depression and psychosis. It was not possible given the data to accurately identify precisely for how long participants had been living with a long-term condition. The decision was therefore taken to include all participants who had ever reported having a long-term condition. This had the disadvantage that it may have included some people where the conditions were historical. As the potential for historical conditions will vary according to what the condition is, this may have introduced some bias into the results.

Many of the participants with a long-term condition also had co-morbidities, which may also have impacted their labour market outcomes. Ideally, the analysis would have matched an individual with a given long-term condition and co-morbidities to a similar individual without that long-term condition but with the same co-morbidities. Unfortunately, the large number of potential combinations of co-morbidities made that impossible. Instead, participants were matched on the number of co-morbidities, in the hope that would create samples in similar health but for the presence of the given long-term condition being studied. This may not have been the case, and it is a limitation of the study that the observed effects may be due to systematically different patterns of co-morbidities between people with/without a given long-term condition.

Another weakness is that, while we observe effects such as people with long-term conditions being more likely to leave the labour market, it is not clear to what extent this is due to barriers to working, or differing individual preferences. People with long-term health conditions face hurdles to full labour market participation, yet it is plausible that they also have different leisure-labour trade-offs. For example, early retirement may become more attractive if life expectancy is reduced, or of the condition leads people to anticipate a decline in quality of life. Thus whilst people with long-term conditions may react differently to a labour market shock this does not necessarily imply that interventions designed to equalise their labour market outcomes would benefit everyone in terms of wellbeing or quality of life.

## Conclusion

The main policy conclusion from these results is that people with many disparate long-term health conditions require even greater support in accessing work in a post-COVID-19 world. Interventions could take many forms, for example supported employment, in which people are placed into a job with support systems provided on an ongoing basis [68]. Other options include eHealth interventions based on mindfulness or cognitive behavioural therapy [11], or ergonomic training [69]. The variety of available interventions, and of people with long-term conditions’ experiences and needs, means interventions will require targeting to be effective, and this should be a topic for future research. Such support would have been of benefit to many people even prior to the pandemic [6–11], but their need is even more pressing now. Disruption in labour market outcomes due to a shock can persist for many years [70–72], creating an ongoing need for support.

Future research should investigate further the causes behind the observed differences in outcomes, particularly whether they reflect the preferences of people with long-term conditions or not. In turn, this could inform research about how best to target labour market interventions. Different people will have different reasons why their long-term conditions affects their labour market participation. Exploring these reasons is important in knowing what support they need to improve their wellbeing.

The studied time period was until September 2021. Yet the impact of COVID-19 on labour market outcomes is still evolving, both on the demand and supply side, for example in the development of new ways of working according to employer and employee priorities [16,73–75]. Future research could assess the longer-term impact of health conditions, including long COVID-19, on labour market outcomes. A further possibility is to investigate how partners of people with long-term conditions, especially those providing informal care, were affected [76].

## Supporting information

S1 Checklist (DOCX)

S1 Table Long-term conditions recorded in understanding society and how classified in current study. (DOCX)

S2 Table Asthma Mahalanobis distance matching for COVID-19 data. (DOCX)

S3 Table Arthritis Mahalanobis distance matching for COVID-19 data. (DOCX)

S4 Table Cancer Mahalanobis distance matching for COVID-19 data. (DOCX)

S5 Table Diabetes Mahalanobis distance matching for COVID-19 data. (DOCX)

S6 Table Emotional, nervous or psychiatric problem Mahalanobis distance matching for COVID-19 data. (DOCX)

S7 Table Vascular conditions Mahalanobis distance matching for COVID-19 data. (DOCX)

S8 Table Pulmonary conditions Mahalanobis distance matching for COVID-19 data. (DOCX)

S9 Table Liver conditions Mahalanobis distance matching for COVID-19 data. (DOCX)

S10 Table Epilepsy Mahalanobis distance matching for COVID-19 data. (DOCX)

S11 Table COVID-19 analysis employment results. (DOCX)

S12 Table COVID-19 analysis hours worked conditional on employment results. (DOCX)

S13 Table COVID-19 analysis furlough results table. (DOCX)

S14 Table COVID-19 analysis working from home results. (DOCX)

S15 Table COVID-19 analysis earnings conditional on employment results. (DOCX)

S16 Table Asthma Mahalanobis distance matching for pre-COVID-19 data. (DOCX)

S17 Table Arthritis Mahalanobis distance matching for pre-COVID-19 data. (DOCX)

S18 Table Cancer Mahalanobis distance matching for pre-COVID-19 data. (DOCX)

S19 Table Diabetes Mahalanobis score matching for pre-COVID-19 data. (DOCX)

S20 Table Emotional, nervous or psychiatric problem Mahalanobis distance score matching for pre-COVID-19 data. (DOCX)

S21 Table Vascular conditions Mahalanobis distance matching for pre-COVID-19 data. (DOCX)

S22 Table Pulmonary conditions Mahalanobis distance matching for pre-COVID-19 data. (DOCX)

S23 Table Liver conditions Mahalanobis distance matching for pre-COVID-19 data. (DOCX)

S24 Table Epilepsy Mahalanobis distance matching for COVID-19 pre-COVID-19 data. (DOCX)

S25 Table Pre-COVID-19 analysis employment results. (DOCX)

S26 Table Pre-COVID-19 analysis hours worked conditional on employment results. (DOCX)

S27 Table Pre-COVID-19 analysis earnings conditional on employment results. (DOCX)
