# Supplementary material for: Long-term health conditions and UK labour market outcomes during the COVID-19 pandemic
Source: PLoS One. 2024 May 10;19(5):e0302746. doi: 10.1371/journal.pone.0302746 (PMC11086911; doi:10.1371/journal.pone.0302746)
Supplement: S1 Table — (DOCX) [file pone.0302746.s002.docx]

**Table S1. Long-term conditions recorded in Understanding Society and how classified in current study.**

| Understanding Society condition definition | Classification in current paper |
| --- | --- |
| Asthma | Asthma |
| Arthritis | Arthritis |
| Congestive heart failure | Vascular |
| Coronary heart disease | Vascular |
| Angina | Vascular |
| Heart attack or myocardial infarction | Vascular |
| Stroke | Vascular |
| Emphysema | Pulmonary |
| Hyperthyroidism or an over-active thyroid | Excluded - thought unlikely to impact labour market outcomes |
| Hypothyroidism or an under-active thyroid | Excluded - thought unlikely to impact labour market outcomes |
| Chronic bronchitis | Pulmonary |
| Any kind of liver condition | Liver |
| Cancer or malignancy | Cancer |
| Diabetes | Diabetes |
| Epilepsy | Epliepsy |
| High blood pressure | Vascular |
| Clinical depression | Emotional, nervous or psychiatric problem |
| Multiple Sclerosis | Excluded - small sample size |
| H.I.V. | Excluded - small sample size |
| COPD (Chronic Obstructive Pulmonary Disease) | Pulmonary |
| An emotional, nervous or psychiatric problem | Emotional, nervous or psychiatric problem |
| Osteoarthritis | Arthritis |
| Rheumatoid arthritis | Arthritis |
| Other arthritis | Arthritis |
| Bowel/colorectal cancer | Cancer |
| Lung cancer | Cancer |
| Breast cancer | Cancer |
| Prostate cancer | Cancer |
| Liver cancer | Cancer |
| Skin cancer or melanoma | Cancer |
| Other cancer | Cancer |
| Type 1 diabetes | Diabetes |
| Type 2 diabetes | Diabetes |
| Gestational diabetes/during pregnancy | Diabetes |
| Other diabetes | Diabetes |
| Anxiety | Emotional, nervous or psychiatric problem |
| Depression | Emotional, nervous or psychiatric problem |
| Psychosis or schizophrenia | Emotional, nervous or psychiatric problem |
| Bipolar disorder or manic depression | Emotional, nervous or psychiatric problem |
| Eating disorder | Emotional, nervous or psychiatric problem |
| Post-traumatic stress disorder | Emotional, nervous or psychiatric problem |
| Other emotional, nervous or psychiatric problem | Emotional, nervous or psychiatric problem |
