# Supplementary material for: Long-term health conditions and UK labour market outcomes during the COVID-19 pandemic
Source: PLoS One. 2024 May 10;19(5):e0302746. doi: 10.1371/journal.pone.0302746 (PMC11086911; doi:10.1371/journal.pone.0302746)
Supplement: S2 Table — (DOCX) [file pone.0302746.s003.docx]

**Table S2. Asthma Mahalanobis distance matching for COVID-19 data.**

|  |  | Treatment | | Control | | SMD |
| --- | --- | --- | --- | --- | --- | --- |
|  |  | N | % | N | % |  |
| Age | mean (sd) | 48.4 | 12.8 | 44.6 | 12.4 | 0.301 |
| Female |  | 3068 | 60.1 | 3041 | 59.6 | 0.0108 |
| White |  | 4468 | 87.5 | 4462 | 87.4 | 3.56x10^-3 |
| Baseline hours worked | mean (sd) | 32.9 | 13.5 | 33.4 | 12.3 | -0.0392 |
| Baseline earnings | mean (sd) | 21.5 | 18.3 | 22.1 | 17.8 | -0.0324 |
| Baseline working from home | always | 338 | 6.6 | 306 | 6 | -0.0327 |
|  | hybrid | 1368 | 26.8 | 1352 | 26.5 |  |
|  | never | 3398 | 66.6 | 3446 | 67.5 |  |
| Key-worker |  | 2217 | 43.4 | 2211 | 43.3 | 2.37x10^-3 |
| Job class | professional | 2380 | 46.6 | 2457 | 48.1 | 0.0275 |
|  | intermediate | 1225 | 24 | 1191 | 23.3 |  |
|  | routine | 1499 | 29.4 | 1456 | 28.5 |  |
| Location | North East | 187 | 3.7 | 145 | 2.8 | -0.0167 |
|  | North West | 514 | 10.1 | 485 | 9.5 |  |
|  | Yorkshire | 439 | 8.6 | 452 | 8.9 |  |
|  | East Midlands | 395 | 7.7 | 400 | 7.8 |  |
|  | West Midlands | 415 | 8.1 | 446 | 8.7 |  |
|  | East England | 482 | 9.4 | 483 | 9.5 |  |
|  | South East | 714 | 14 | 657 | 12.9 |  |
|  | South West | 487 | 9.5 | 472 | 9.2 |  |
|  | London | 544 | 10.7 | 614 | 12 |  |
|  | Wales | 296 | 5.8 | 309 | 6.1 |  |
|  | Scotland | 424 | 8.3 | 431 | 8.4 |  |
|  | Northern Ireland | 207 | 4.1 | 210 | 4.1 |  |
| Household size | mean (sd) | 2.9 | 1.2 | 3 | 1.1 | -0.136 |
| Baseline household income | mean (sd) | 37.3 | 29 | 39.2 | 26 | -0.0641 |
| Baseline receiving UC |  | 108 | 2.1 | 108 | 2.1 | 0 |
| Number of comorbidities | mean (sd) | 1.6 | 1.5 | 0.4 | 0.7 | 0.837 |
| N |  | 5104 |  | 5104 |  |  |
| *Note.* SMD=standardised mean difference; UC=universal credit | | | | | | |
