# Supplementary material for: Long-term health conditions and UK labour market outcomes during the COVID-19 pandemic
Source: PLoS One. 2024 May 10;19(5):e0302746. doi: 10.1371/journal.pone.0302746 (PMC11086911; doi:10.1371/journal.pone.0302746)
Supplement: S3 Table — (DOCX) [file pone.0302746.s004.docx]

**Table S3. Arthritis Mahalanobis distance matching for COVID-19 data.**

|  |  | Treatment | | Control | | SMD |
| --- | --- | --- | --- | --- | --- | --- |
|  |  | N | % | N | % |  |
| Age | mean (sd) | 52.8 | 11.7 | 50.1 | 11.3 | 0.234 |
| Female |  | 1463 | 63.5 | 1461 | 63.4 | 1.80x10^-3 |
| White |  | 2052 | 89.1 | 2052 | 89.1 | 0 |
| Baseline hours worked | mean (sd) | 32.2 | 14.2 | 32.3 | 13 | -9.83x10^-3 |
| Baseline earnings | mean (sd) | 20.1 | 16.2 | 20.3 | 15.5 | -0.014 |
| Baseline working from home | always | 181 | 7.9 | 167 | 7.2 | -0.0217 |
|  | hybrid | 567 | 24.6 | 571 | 24.8 |  |
|  | never | 1556 | 67.5 | 1566 | 68 |  |
| Key-worker |  | 1032 | 44.8 | 1031 | 44.7 | 8.73x10^-4 |
| Job class | professional | 986 | 42.8 | 1023 | 44.4 | 0.0263 |
|  | intermediate | 586 | 25.4 | 564 | 24.5 |  |
|  | routine | 732 | 31.8 | 717 | 31.1 |  |
| Location | North East | 94 | 4.1 | 61 | 2.6 | -0.0219 |
|  | North West | 236 | 10.2 | 221 | 9.6 |  |
|  | Yorkshire | 180 | 7.8 | 214 | 9.3 |  |
|  | East Midlands | 192 | 8.3 | 175 | 7.6 |  |
|  | West Midlands | 195 | 8.5 | 207 | 9 |  |
|  | East England | 221 | 9.6 | 214 | 9.3 |  |
|  | South East | 320 | 13.9 | 326 | 14.1 |  |
|  | South West | 232 | 10.1 | 223 | 9.7 |  |
|  | London | 220 | 9.5 | 254 | 11 |  |
|  | Wales | 146 | 6.3 | 134 | 5.8 |  |
|  | Scotland | 181 | 7.9 | 184 | 8 |  |
|  | Northern Ireland | 87 | 3.8 | 91 | 3.9 |  |
| Household size | mean (sd) | 2.7 | 1.2 | 2.8 | 1.1 | -0.104 |
| Baseline household income | mean (sd) | 34.4 | 26.8 | 35.4 | 23.6 | -0.0343 |
| Baseline receiving UC |  | 55 | 2.4 | 55 | 2.4 | 0 |
| Number of comorbidities | mean (sd) | 2.5 | 1.7 | 1.8 | 1.2 | 0.448 |
| N |  | 2304 |  | 2304 |  |  |
| *Note.* SMD=standardised mean difference; UC=universal credit | | | | | | |
