# Supplementary material for: Long-term health conditions and UK labour market outcomes during the COVID-19 pandemic
Source: PLoS One. 2024 May 10;19(5):e0302746. doi: 10.1371/journal.pone.0302746 (PMC11086911; doi:10.1371/journal.pone.0302746)
Supplement: S4 Table — (DOCX) [file pone.0302746.s005.docx]

**Table S4. Cancer Mahalanobis distance matching for COVID-19 data.**

|  |  | Treatment | | Control | | SMD |
| --- | --- | --- | --- | --- | --- | --- |
|  |  | N | % | N | % |  |
| Age | mean (sd) | 55.9 | 10.5 | 54.2 | 10.2 | 0.169 |
| Female |  | 363 | 65.6 | 727 | 65.7 | -1.90x10^-3 |
| White |  | 519 | 93.9 | 1039 | 93.9 | -3.76x10^-3 |
| Baseline hours worked | mean (sd) | 30.3 | 14.3 | 30.2 | 12.9 | 2.69x10^-3 |
| Baseline earnings | mean (sd) | 21.5 | 20.4 | 20.6 | 18.8 | 0.043 |
| Baseline working from home | always | 54 | 9.8 | 108 | 9.8 | -0.0243 |
|  | hybrid | 149 | 26.9 | 287 | 25.9 |  |
|  | never | 350 | 63.3 | 711 | 64.3 |  |
| Key-worker |  | 208 | 37.6 | 408 | 36.9 | 0.0149 |
| Job class | professional | 273 | 49.4 | 541 | 48.9 | -9.89x10^-3 |
|  | intermediate | 145 | 26.2 | 291 | 26.3 |  |
|  | routine | 135 | 24.4 | 274 | 24.8 |  |
| Location | North East | 16 | 2.9 | 23 | 2.1 | 5.15x10^-3 |
|  | North West | 52 | 9.4 | 102 | 9.2 |  |
|  | Yorkshire | 43 | 7.8 | 91 | 8.2 |  |
|  | East Midlands | 43 | 7.8 | 96 | 8.7 |  |
|  | West Midlands | 46 | 8.3 | 83 | 7.5 |  |
|  | East England | 51 | 9.2 | 107 | 9.7 |  |
|  | South East | 92 | 16.6 | 182 | 16.5 |  |
|  | South West | 72 | 13 | 113 | 10.2 |  |
|  | London | 53 | 9.6 | 120 | 10.8 |  |
|  | Wales | 24 | 4.3 | 76 | 6.9 |  |
|  | Scotland | 34 | 6.1 | 87 | 7.9 |  |
|  | Northern Ireland | 27 | 4.9 | 26 | 2.4 |  |
| Household size | mean (sd) | 2.6 | 1.2 | 2.6 | 1.1 | -9.06x10^-3 |
| Baseline household income | mean (sd) | 36.7 | 27.5 | 35.1 | 24.7 | 0.058 |
| Baseline receiving UC |  | 11 | 2 | 22 | 2 | 0 |
| Number of comorbidities | mean (sd) | 2.1 | 2 | 1.8 | 1.8 | 0.128 |
| N |  | 553 |  | 1106 |  |  |
| *Note.* SMD=standardised mean difference; UC=universal credit | | | | | | |
