# Supplementary material for: Long-term health conditions and UK labour market outcomes during the COVID-19 pandemic
Source: PLoS One. 2024 May 10;19(5):e0302746. doi: 10.1371/journal.pone.0302746 (PMC11086911; doi:10.1371/journal.pone.0302746)
Supplement: S5 Table — (DOCX) [file pone.0302746.s006.docx]

**Table S5. Diabetes Mahalanobis distance matching for COVID-19 data.**

|  |  | Treatment | | Control | | SMD |
| --- | --- | --- | --- | --- | --- | --- |
|  |  | N | % | N | % |  |
| Age | mean (sd) | 53.5 | 10.7 | 51.6 | 10.3 | 0.18 |
| Female |  | 278 | 46 | 560 | 46.4 | -6.64x10^-3 |
| White |  | 455 | 75.3 | 910 | 75.3 | 0 |
| Baseline hours worked | mean (sd) | 32.1 | 12.9 | 32.3 | 11.5 | -0.0181 |
| Baseline earnings | mean (sd) | 21.5 | 16.7 | 21 | 15.3 | 0.0309 |
| Baseline working from home | always | 51 | 8.4 | 93 | 7.7 | -0.0425 |
|  | hybrid | 163 | 27 | 311 | 25.7 |  |
|  | never | 390 | 64.6 | 804 | 66.6 |  |
| Key-worker |  | 254 | 42.1 | 512 | 42.4 | -6.71x10^-3 |
| Job class | professional | 263 | 43.5 | 523 | 43.3 | -9.78x10^-3 |
|  | intermediate | 161 | 26.7 | 318 | 26.3 |  |
|  | routine | 180 | 29.8 | 367 | 30.4 |  |
| Location | North East | 19 | 3.1 | 36 | 3 | 0.0113 |
|  | North West | 58 | 9.6 | 101 | 8.4 |  |
|  | Yorkshire | 44 | 7.3 | 109 | 9 |  |
|  | East Midlands | 42 | 7 | 85 | 7 |  |
|  | West Midlands | 61 | 10.1 | 96 | 7.9 |  |
|  | East England | 53 | 8.8 | 108 | 8.9 |  |
|  | South East | 88 | 14.6 | 172 | 14.2 |  |
|  | South West | 47 | 7.8 | 118 | 9.8 |  |
|  | London | 92 | 15.2 | 209 | 17.3 |  |
|  | Wales | 35 | 5.8 | 76 | 6.3 |  |
|  | Scotland | 40 | 6.6 | 72 | 6 |  |
|  | Northern Ireland | 25 | 4.1 | 26 | 2.2 |  |
| Household size | mean (sd) | 2.9 | 1.3 | 2.9 | 1.2 | -3.31x10^-3 |
| Baseline household income | mean (sd) | 33.6 | 23.4 | 34.3 | 22.2 | -0.0297 |
| Baseline receiving UC |  | 15 | 2.5 | 30 | 2.5 | 0 |
| Number of comorbidities | mean (sd) | 2.8 | 2 | 2.3 | 1.9 | 0.255 |
| N |  | 604 |  | 1208 |  |  |
| *Note.* SMD=standardised mean difference; UC=universal credit | | | | | | |
