# Supplementary material for: Long-term health conditions and UK labour market outcomes during the COVID-19 pandemic
Source: PLoS One. 2024 May 10;19(5):e0302746. doi: 10.1371/journal.pone.0302746 (PMC11086911; doi:10.1371/journal.pone.0302746)
Supplement: S6 Table — (DOCX) [file pone.0302746.s007.docx]

**Table S6. Emotional, nervous or psychiatric problem Mahalanobis distance matching for COVID-19 data.**

|  |  | Treatment | | Control | | SMD |
| --- | --- | --- | --- | --- | --- | --- |
|  |  | N | % | N | % |  |
| Age | mean (sd) | 45.2 | 12.9 | 45.4 | 12.7 | -0.0106 |
| Female |  | 1088 | 71.7 | 1086 | 71.6 | 2.93x10^-3 |
| White |  | 1400 | 92.3 | 1400 | 92.3 | 0 |
| Baseline hours worked | mean (sd) | 31.9 | 13 | 31.9 | 12 | -7.89x10^-4 |
| Baseline earnings | mean (sd) | 19.4 | 15.8 | 19.7 | 15.4 | -0.0244 |
| Baseline working from home | always | 101 | 6.7 | 87 | 5.7 | -0.0347 |
|  | hybrid | 374 | 24.7 | 371 | 24.5 |  |
|  | never | 1042 | 68.7 | 1059 | 69.8 |  |
| Key-worker |  | 731 | 48.2 | 730 | 48.1 | 1.32x10^-3 |
| Job class | professional | 681 | 44.9 | 691 | 45.6 | 9.20x10^-3 |
|  | intermediate | 366 | 24.1 | 358 | 23.6 |  |
|  | routine | 470 | 31 | 468 | 30.9 |  |
| Location | North East | 66 | 4.4 | 39 | 2.6 | -2.32x10^-3 |
|  | North West | 151 | 10 | 165 | 10.9 |  |
|  | Yorkshire | 128 | 8.4 | 127 | 8.4 |  |
|  | East Midlands | 113 | 7.4 | 133 | 8.8 |  |
|  | West Midlands | 118 | 7.8 | 120 | 7.9 |  |
|  | East England | 157 | 10.3 | 132 | 8.7 |  |
|  | South East | 208 | 13.7 | 203 | 13.4 |  |
|  | South West | 155 | 10.2 | 146 | 9.6 |  |
|  | London | 140 | 9.2 | 166 | 10.9 |  |
|  | Wales | 82 | 5.4 | 115 | 7.6 |  |
|  | Scotland | 138 | 9.1 | 120 | 7.9 |  |
|  | Northern Ireland | 61 | 4 | 51 | 3.4 |  |
| Household size | mean (sd) | 2.8 | 1.2 | 2.9 | 1.1 | -0.0358 |
| Baseline household income | mean (sd) | 35.2 | 28.8 | 35.9 | 24.4 | -0.0242 |
| Baseline receiving UC |  | 54 | 3.6 | 54 | 3.6 | 0 |
| Number of comorbidities | mean (sd) | 2.2 | 1.8 | 1.8 | 1.6 | 0.216 |
| N |  | 1517 |  | 1517 |  |  |
| *Note.* SMD=standardised mean difference; UC=universal credit | | | | | | |
