# Supplementary material for: Long-term health conditions and UK labour market outcomes during the COVID-19 pandemic
Source: PLoS One. 2024 May 10;19(5):e0302746. doi: 10.1371/journal.pone.0302746 (PMC11086911; doi:10.1371/journal.pone.0302746)
Supplement: S7 Table — (DOCX) [file pone.0302746.s008.docx]

**Table S7. Vascular conditions Mahalanobis distance matching for COVID-19 data.**

|  |  | Treatment | | Control | | SMD |
| --- | --- | --- | --- | --- | --- | --- |
|  |  | N | % | N | % |  |
| Age | mean (sd) | 53.8 | 10.8 | 51 | 10.6 | 0.264 |
| Female |  | 1162 | 51.9 | 1177 | 52.5 | -0.0134 |
| White |  | 1962 | 87.6 | 1964 | 87.6 | -2.70x10^-3 |
| Baseline hours worked | mean (sd) | 32.9 | 14.1 | 33.2 | 13.1 | -0.0188 |
| Baseline earnings | mean (sd) | 22 | 20 | 22.3 | 19.6 | -0.0138 |
| Baseline working from home | always | 181 | 8.1 | 160 | 7.1 | -0.0281 |
|  | hybrid | 574 | 25.6 | 584 | 26.1 |  |
|  | never | 1486 | 66.3 | 1497 | 66.8 |  |
| Key-worker |  | 932 | 41.6 | 927 | 41.4 | 4.53x10^-3 |
| Job class | professional | 1012 | 45.2 | 1039 | 46.4 | 0.0163 |
|  | intermediate | 573 | 25.6 | 550 | 24.5 |  |
|  | routine | 656 | 29.3 | 652 | 29.1 |  |
| Location | North East | 76 | 3.4 | 65 | 2.9 | -7.43x10^-4 |
|  | North West | 219 | 9.8 | 206 | 9.2 |  |
|  | Yorkshire | 169 | 7.5 | 198 | 8.8 |  |
|  | East Midlands | 165 | 7.4 | 176 | 7.9 |  |
|  | West Midlands | 196 | 8.7 | 184 | 8.2 |  |
|  | East England | 216 | 9.6 | 216 | 9.6 |  |
|  | South East | 322 | 14.4 | 305 | 13.6 |  |
|  | South West | 199 | 8.9 | 258 | 11.5 |  |
|  | London | 276 | 12.3 | 259 | 11.6 |  |
|  | Wales | 138 | 6.2 | 123 | 5.5 |  |
|  | Scotland | 188 | 8.4 | 175 | 7.8 |  |
|  | Northern Ireland | 77 | 3.4 | 76 | 3.4 |  |
| Household size | mean (sd) | 2.7 | 1.2 | 2.8 | 1.1 | -0.0867 |
| Baseline household income | mean (sd) | 35.4 | 27.4 | 35.9 | 24.6 | -0.0159 |
| Baseline receiving UC |  | 47 | 2.1 | 47 | 2.1 | 0 |
| Number of comorbidities | mean (sd) | 2.3 | 1.8 | 1.7 | 1.3 | 0.361 |
| N |  | 2241 |  | 2241 |  |  |
| *Note.* SMD=standardised mean difference; UC=universal credit | | | | | | |
