# Supplementary material for: Long-term health conditions and UK labour market outcomes during the COVID-19 pandemic
Source: PLoS One. 2024 May 10;19(5):e0302746. doi: 10.1371/journal.pone.0302746 (PMC11086911; doi:10.1371/journal.pone.0302746)
Supplement: S8 Table — (DOCX) [file pone.0302746.s009.docx]

**Table S8. Pulmonary conditions Mahalanobis distance matching for COVID-19 data.**

|  |  | Treatment | | Control | | SMD |
| --- | --- | --- | --- | --- | --- | --- |
|  |  | N | % | N | % |  |
| Age | mean (sd) | 56.1 | 10.6 | 54 | 10.1 | 0.194 |
| Female |  | 112 | 56.3 | 339 | 56.8 | -0.0101 |
| White |  | 188 | 94.5 | 563 | 94.3 | 7.33x10^-3 |
| Baseline hours worked | mean (sd) | 32.3 | 12.7 | 32.7 | 11.3 | -0.0245 |
| Baseline earnings | mean (sd) | 19.4 | 17.8 | 19.6 | 16.9 | -0.0107 |
| Baseline working from home | always | 16 | 8 | 50 | 8.4 | -0.0349 |
|  | hybrid | 47 | 23.6 | 130 | 21.8 |  |
|  | never | 136 | 68.3 | 417 | 69.8 |  |
| Key-worker |  | 92 | 46.2 | 276 | 46.2 | -5.55x10^-17 |
| Job class | professional | 83 | 41.7 | 240 | 40.2 | -0.0152 |
|  | intermediate | 44 | 22.1 | 142 | 23.8 |  |
|  | routine | 72 | 36.2 | 215 | 36 |  |
| Location | North East | 4 | 2 | 10 | 1.7 | -0.0373 |
|  | North West | 25 | 12.6 | 53 | 8.9 |  |
|  | Yorkshire | 16 | 8 | 48 | 8 |  |
|  | East Midlands | 17 | 8.5 | 59 | 9.9 |  |
|  | West Midlands | 10 | 5 | 40 | 6.7 |  |
|  | East England | 21 | 10.6 | 66 | 11.1 |  |
|  | South East | 26 | 13.1 | 88 | 14.7 |  |
|  | South West | 30 | 15.1 | 65 | 10.9 |  |
|  | London | 15 | 7.5 | 48 | 8 |  |
|  | Wales | 12 | 6 | 54 | 9 |  |
|  | Scotland | 15 | 7.5 | 50 | 8.4 |  |
|  | Northern Ireland | 8 | 4 | 16 | 2.7 |  |
| Household size | mean (sd) | 2.4 | 1 | 2.5 | 1 | -0.0676 |
| Baseline household income | mean (sd) | 30.3 | 24.8 | 31.7 | 22 | -0.0576 |
| Baseline receiving UC |  | 5 | 2.5 | 15 | 2.5 | 0 |
| Number of comorbidities | mean (sd) | 3.4 | 2.3 | 2.9 | 2 | 0.208 |
| N |  | 199 |  | 597 |  |  |
| *Note.* SMD=standardised mean difference; UC=universal credit | | | | | | |
