# Supplementary material for: Long-term health conditions and UK labour market outcomes during the COVID-19 pandemic
Source: PLoS One. 2024 May 10;19(5):e0302746. doi: 10.1371/journal.pone.0302746 (PMC11086911; doi:10.1371/journal.pone.0302746)
Supplement: S9 Table — (DOCX) [file pone.0302746.s010.docx]

**Table S9. Liver conditions Mahalanobis distance matching for COVID-19 data.**

|  |  | Treatment | | Control | | SMD |
| --- | --- | --- | --- | --- | --- | --- |
|  |  | N | % | N | % |  |
| Age | mean (sd) | 50.6 | 11.3 | 49.7 | 11.4 | 0.085 |
| Female |  | 132 | 52.6 | 395 | 52.5 | 2.66x10^-3 |
| White |  | 223 | 88.8 | 669 | 88.8 | 0 |
| Baseline hours worked | mean (sd) | 32 | 12.8 | 32.3 | 11.7 | -0.018 |
| Baseline earnings | mean (sd) | 21.2 | 14.4 | 20.9 | 13.8 | 0.0175 |
| Baseline working from home | always | 19 | 7.6 | 52 | 6.9 | -0.0542 |
|  | hybrid | 79 | 31.5 | 216 | 28.7 |  |
|  | never | 153 | 61 | 485 | 64.4 |  |
| Key-worker |  | 105 | 41.8 | 316 | 42 | -2.69x10^-3 |
| Job class | professional | 129 | 51.4 | 389 | 51.7 | -4.67x10^-3 |
|  | intermediate | 54 | 21.5 | 155 | 20.6 |  |
|  | routine | 68 | 27.1 | 209 | 27.8 |  |
| Location | North East | 11 | 4.4 | 18 | 2.4 | 7.31x10^-3 |
|  | North West | 27 | 10.8 | 78 | 10.4 |  |
|  | Yorkshire | 14 | 5.6 | 71 | 9.4 |  |
|  | East Midlands | 20 | 8 | 49 | 6.5 |  |
|  | West Midlands | 20 | 8 | 63 | 8.4 |  |
|  | East England | 26 | 10.4 | 72 | 9.6 |  |
|  | South East | 35 | 13.9 | 121 | 16.1 |  |
|  | South West | 27 | 10.8 | 77 | 10.2 |  |
|  | London | 25 | 10 | 77 | 10.2 |  |
|  | Wales | 16 | 6.4 | 47 | 6.2 |  |
|  | Scotland | 20 | 8 | 54 | 7.2 |  |
|  | Northern Ireland | 10 | 4 | 26 | 3.5 |  |
| Household size | mean (sd) | 2.8 | 1.2 | 2.8 | 1.1 | -0.0314 |
| Baseline household income | mean (sd) | 35.6 | 27 | 35.8 | 24.7 | -8.49x10^-3 |
| Baseline receiving UC |  | 7 | 2.8 | 21 | 2.8 | 0 |
| Number of comorbidities | mean (sd) | 2.9 | 2.1 | 2.5 | 1.9 | 0.21 |
| N |  | 251 |  | 753 |  |  |
| *Note.* SMD=standardised mean difference; UC=universal credit | | | | | | |
