# Supplementary material for: Long-term health conditions and UK labour market outcomes during the COVID-19 pandemic
Source: PLoS One. 2024 May 10;19(5):e0302746. doi: 10.1371/journal.pone.0302746 (PMC11086911; doi:10.1371/journal.pone.0302746)
Supplement: S10 Table — (DOCX) [file pone.0302746.s011.docx]

**Table S10. Epilepsy Mahalanobis distance matching for COVID-19 data.**

|  |  | Treatment | | Control | | SMD |
| --- | --- | --- | --- | --- | --- | --- |
|  |  | N | % | N | % |  |
| Age | mean (sd) | 46 | 11.9 | 45.7 | 12 | 0.0323 |
| Female |  | 69 | 53.1 | 280 | 53.8 | -0.0154 |
| White |  | 121 | 93.1 | 484 | 93.1 | 0 |
| Baseline hours worked | mean (sd) | 33.1 | 13.6 | 33.1 | 11.9 | 1.40x10^-3 |
| Baseline earnings | mean (sd) | 23 | 22.9 | 22.5 | 21.7 | 0.0244 |
| Baseline working from home | always | 6 | 4.6 | 21 | 4 | -0.0409 |
|  | hybrid | 35 | 26.9 | 136 | 26.2 |  |
|  | never | 89 | 68.5 | 363 | 69.8 |  |
| Key-worker |  | 54 | 41.5 | 215 | 41.3 | 3.90x10^-3 |
| Job class | professional | 59 | 45.4 | 238 | 45.8 | -8.58x10^-3 |
|  | intermediate | 25 | 19.2 | 92 | 17.7 |  |
|  | routine | 46 | 35.4 | 190 | 36.5 |  |
| Location | North East | 7 | 5.4 | 7 | 1.3 | -0.0124 |
|  | North West | 5 | 3.8 | 33 | 6.3 |  |
|  | Yorkshire | 8 | 6.2 | 53 | 10.2 |  |
|  | East Midlands | 12 | 9.2 | 36 | 6.9 |  |
|  | West Midlands | 9 | 6.9 | 45 | 8.7 |  |
|  | East England | 17 | 13.1 | 57 | 11 |  |
|  | South East | 16 | 12.3 | 92 | 17.7 |  |
|  | South West | 14 | 10.8 | 56 | 10.8 |  |
|  | London | 17 | 13.1 | 43 | 8.3 |  |
|  | Wales | 7 | 5.4 | 33 | 6.3 |  |
|  | Scotland | 13 | 10 | 47 | 9 |  |
|  | Northern Ireland | 5 | 3.8 | 18 | 3.5 |  |
| Household size | mean (sd) | 2.8 | 1.1 | 2.8 | 1.1 | -0.0422 |
| Baseline household income | mean (sd) | 36.4 | 23.2 | 36 | 22 | 0.0172 |
| Baseline receiving UC |  | 1 | 0.8 | 4 | 0.8 | 0 |
| Number of comorbidities | mean (sd) | 2.1 | 2.1 | 1.8 | 2 | 0.15 |
| N |  | 130 |  | 520 |  |  |
| *Note.* SMD=standardised mean difference; UC=universal credit | | | | | | |
