# Supplementary material for: Long-term health conditions and UK labour market outcomes during the COVID-19 pandemic
Source: PLoS One. 2024 May 10;19(5):e0302746. doi: 10.1371/journal.pone.0302746 (PMC11086911; doi:10.1371/journal.pone.0302746)
Supplement: S11 Table — (DOCX) [file pone.0302746.s012.docx]

**Table S11. COVID-19 analysis employment results.**

|  | Asthma | | Arthritis | | Cancer | | Diabetes | | ENP | | Vascular | | Pulmonary | | Liver | | Epilepsy | |
| --- | --- | --- | --- | --- | --- | --- | --- | --- | --- | --- | --- | --- | --- | --- | --- | --- | --- | --- |
|  | Coeff. | *p* | Coeff. | *p* | Coeff. | *p* | Coeff. | *p* | Coeff. | *p* | Coeff. | *p* | Coeff. | *p* | Coeff. | *p* | Coeff. | *p* |
| LTC | -0.143 | 0.415 | -0.0338 | 0.876 | -0.437 | 0.18 | 0.717 | 0.088 | -0.648 | 0.017* | 0.195 | 0.406 | 0.394 | 0.408 | -1.07 | 0.010* | -0.473 | 0.474 |
| *t* | -0.0952 | 0.000* | -0.135 | 0.000* | -0.172 | 0.000* | -0.16 | 0.000* | -0.114 | 0.000* | -0.142 | 0.000* | -0.185 | 0.000* | -0.106 | 0.000* | -0.0545 | 0.015* |
| LTC × *t* | -0.0406 | 0.000* | -0.032 | 0.019* | -0.0278 | 0.231 | -0.0427 | 0.075 | 0.0111 | 0.513 | -0.0359 | 0.013* | -0.0157 | 0.71 | 9.61x10^-3 | 0.777 | -0.19 | 0.000* |
| ln age | -0.385 | 0.103 | -1.35 | 0.000* | -2.24 | 0.003* | -4.25 | 0.000* | -0.177 | 0.637 | -1.77 | 0.000* | -6.11 | 0.000* | -1.32 | 0.041* | 0.81 | 0.395 |
| Female | -0.0531 | 0.739 | -0.0175 | 0.936 | -0.229 | 0.424 | 0.0377 | 0.924 | -0.0751 | 0.777 | -0.0406 | 0.857 | -0.834 | 0.017* | 0.671 | 0.033* | 0.0955 | 0.855 |
| White | 1.64 | 0.000* | 1.11 | 0.001* | 1.26 | 0.002* | 1.87 | 0.000* | 1 | 0.096 | 1.52 | 0.000* | 0.968 | 0.268 | 2.31 | 0.000* | 2.57 | 0.001* |
| Household size | 0.186 | 0.000* | 0.318 | 0.000* | 0.25 | 0.037* | 0.138 | 0.242 | 0.229 | 0.013* | 0.378 | 0.000* | 0.167 | 0.356 | 0.0878 | 0.499 | 0.186 | 0.265 |
| Baseline hours worked | 0.0722 | 0.000* | 0.0647 | 0.000* | 0.0757 | 0.000* | 0.0718 | 0.000* | 0.049 | 0.000* | 0.07 | 0.000* | 0.127 | 0.000* | 0.113 | 0.000* | 0.0279 | 0.208 |
| Baseline earnings | 5.52x10^-3 | 0.31 | -3.08x10^-3 | 0.697 | -0.0217 | 0.035* | -3.27x10^-3 | 0.812 | -5.34x10^-4 | 0.952 | 2.64x10^-3 | 0.765 | -0.0366 | 0.039* | -0.0174 | 0.152 | 6.90x10^-3 | 0.659 |
| Baseline household income | 8.68x10^-3 | 0.010* | 0.0122 | 0.014* | 0.0257 | 0.000* | 8.22x10^-3 | 0.376 | 8.98x10^-3 | 0.11 | 0.0112 | 0.039* | 0.0357 | 0.008* | -5.38x10^-4 | 0.947 | 3.34x10^-3 | 0.774 |
| Baseline work from home - hybrid | 1.01 | 0.000* | 1.34 | 0.000* | 2.16 | 0.000* | 0.794 | 0.074 | 1.31 | 0.000* | 1.18 | 0.000* | 0.143 | 0.761 | 1.46 | 0.001* | 1.84 | 0.016* |
| Baseline work from home - always | 0.712 | 0.005* | 0.929 | 0.026* | 1.98 | 0.000* | 0.633 | 0.29 | 1.18 | 0.028* | 0.772 | 0.033* | 2.09 | 0.004* | -0.895 | 0.054 | 1.16 | 0.361 |
| Location - North East | 0.537 | 0.284 | 0.0883 | 0.876 | 3.6 | 0.037* | 0.358 | 0.775 | 0.848 | 0.186 | -0.612 | 0.307 | 13.5 | 0.584 | 0.955 | 0.368 | -1.61 | 0.299 |
| Location - North West | 0.16 | 0.651 | 0.049 | 0.92 | 1.28 | 0.064 | -0.346 | 0.646 | -0.55 | 0.312 | 0.246 | 0.655 | -1.83 | 0.015* | -0.929 | 0.133 | -0.627 | 0.61 |
| Location - Yorkshire | -0.0752 | 0.869 | 0.531 | 0.298 | 1.32 | 0.028* | 0.186 | 0.81 | -0.224 | 0.67 | 0.225 | 0.73 | -0.489 | 0.55 | 0.467 | 0.616 | -2.05 | 0.053 |
| Location - East Midlands | 0.109 | 0.757 | 0.101 | 0.838 | 0.582 | 0.311 | -0.361 | 0.675 | -0.753 | 0.125 | -0.201 | 0.716 | -3.13 | 0.000* | -0.0243 | 0.976 | -1.14 | 0.297 |
| Location - West Midlands | 0.254 | 0.449 | 0.516 | 0.236 | 2.32 | 0.000* | 0.84 | 0.356 | -0.0586 | 0.924 | 0.27 | 0.614 | -1.48 | 0.072 | 0.224 | 0.721 | -0.492 | 0.67 |
| Location - East England | -0.323 | 0.264 | -0.029 | 0.949 | 0.751 | 0.204 | -1.09 | 0.171 | -0.543 | 0.263 | -0.46 | 0.375 | -1.81 | 0.008* | 0.356 | 0.583 | -0.852 | 0.432 |
| Location - South East | -0.0322 | 0.907 | 0.0116 | 0.979 | 0.724 | 0.095 | -0.0347 | 0.961 | -0.358 | 0.384 | -0.652 | 0.193 | -0.849 | 0.232 | 0.349 | 0.573 | -1.12 | 0.278 |
| Location - South West | 0.391 | 0.226 | 0.815 | 0.12 | 1.45 | 0.005* | 1.41 | 0.124 | 0.636 | 0.187 | 0.751 | 0.218 | -0.0799 | 0.931 | 1.27 | 0.055 | -0.83 | 0.419 |
| Location - Wales | -0.305 | 0.389 | 0.474 | 0.38 | 1.57 | 0.009* | -0.0705 | 0.928 | -0.198 | 0.699 | -0.564 | 0.309 | 0.386 | 0.641 | 1.21 | 0.234 | -0.976 | 0.419 |
| Location - Scotland | 0.253 | 0.471 | 0.586 | 0.22 | 1.86 | 0.014* | 1.76 | 0.085 | 0.28 | 0.596 | 0.333 | 0.589 | 1.56 | 0.168 | -0.653 | 0.395 | -1.11 | 0.383 |
| Location - Northern Ireland | 0.186 | 0.616 | 0.403 | 0.496 | 1.47 | 0.023* | -1.04 | 0.395 | 0.806 | 0.36 | 0.132 | 0.83 | 0.393 | 0.729 | 3.75 | 0.093 | -2.54 | 0.039* |
| Number of comorbidities | -0.192 | 0.001* | -0.11 | 0.137 | -0.344 | 0.000* | -0.14 | 0.06 | -0.19 | 0.022* | -0.203 | 0.003* | -0.175 | 0.019* | -0.31 | 0.000* | -0.29 | 0.026* |
| Constant | 2.72 | 0.004* | 6.91 | 0.000* | 10.4 | 0.001* | 18.7 | 0.000* | 3.32 | 0.043* | 8.64 | 0.000* | 27.4 | 0.000* | 7.87 | 0.005* | 0.055 | 0.989 |
| N respondents | 10208 |  | 4608 |  | 1659 |  | 1812 |  | 3034 |  | 4482 |  | 796 |  | 1004 |  | 650 |  |
| N observations | 61865 |  | 29463 |  | 10851 |  | 10894 |  | 18841 |  | 28343 |  | 5108 |  | 6195 |  | 3867 |  |
| *Note.* LTC=Long-term condition; *t*=months after April 2020, ENP=emotional, nervous, or psychiatric problem; Coeff.=coefficient; *=significant at 5% level | | | | | | | | | | | | | | | | | | |
