# Supplementary material for: Long-term health conditions and UK labour market outcomes during the COVID-19 pandemic
Source: PLoS One. 2024 May 10;19(5):e0302746. doi: 10.1371/journal.pone.0302746 (PMC11086911; doi:10.1371/journal.pone.0302746)
Supplement: S12 Table — (DOCX) [file pone.0302746.s013.docx]

**Table S12. COVID-19 analysis hours worked conditional on employment results.**

|  | Asthma | | Arthritis | | Cancer | | Diabetes | | ENP | | Vascular | | Pulmonary | | Liver | | Epilepsy | |
| --- | --- | --- | --- | --- | --- | --- | --- | --- | --- | --- | --- | --- | --- | --- | --- | --- | --- | --- |
|  | Coeff. | *p* | Coeff. | *p* | Coeff. | *p* | Coeff. | *p* | Coeff. | *p* | Coeff. | *p* | Coeff. | *p* | Coeff. | *p* | Coeff. | *p* |
| LTC | -0.0463 | 0.847 | 0.182 | 0.587 | -1.02 | 0.065 | -1.13 | 0.029* | -0.497 | 0.218 | -0.406 | 0.235 | -2.46 | 0.023* | -0.161 | 0.839 | -1.39 | 0.251 |
| *t* | 0.559 | 0.000* | 0.594 | 0.000* | 0.502 | 0.000* | 0.566 | 0.000* | 0.576 | 0.000* | 0.534 | 0.000* | 0.63 | 0.000* | 0.652 | 0.000* | 0.535 | 0.000* |
| LTC × *t* | 7.43x10^-3 | 0.647 | -0.0589 | 0.017* | 0.0672 | 0.086 | 0.0318 | 0.421 | -0.0283 | 0.332 | 0.0145 | 0.57 | -0.0622 | 0.408 | -0.11 | 0.07 | 0.11 | 0.206 |
| ln age | -1.87 | 0.000* | -2.99 | 0.000* | -2.24 | 0.103 | -3.03 | 0.031* | -1.69 | 0.021* | -2.45 | 0.002* | 0.169 | 0.946 | 0.893 | 0.602 | 3.71 | 0.023* |
| Female | -1.13 | 0.000* | -0.36 | 0.295 | 0.448 | 0.435 | -0.24 | 0.659 | -1.1 | 0.014* | -0.555 | 0.116 | 0.142 | 0.886 | 0.0876 | 0.912 | -0.832 | 0.414 |
| White | -0.764 | 0.046* | -0.891 | 0.14 | 0.156 | 0.901 | 0.0186 | 0.98 | -0.0611 | 0.94 | 0.149 | 0.811 | -5.89 | 0.019* | 0.237 | 0.881 | -2.3 | 0.266 |
| Household size | -0.207 | 0.005* | -0.193 | 0.106 | -0.0807 | 0.677 | 0.0206 | 0.916 | -0.0885 | 0.513 | 0.0778 | 0.542 | -0.363 | 0.307 | 0.137 | 0.617 | 0.418 | 0.185 |
| Baseline hours worked | 0.676 | 0.000* | 0.656 | 0.000* | 0.815 | 0.000* | 0.796 | 0.000* | 0.628 | 0.000* | 0.65 | 0.000* | 0.74 | 0.000* | 0.93 | 0.000* | 0.738 | 0.000* |
| Baseline earnings | 0.114 | 0.000* | 0.155 | 0.000* | 0.0665 | 0.010* | 0.115 | 0.000* | 0.139 | 0.000* | 0.105 | 0.000* | 0.0874 | 0.003* | 0.0659 | 0.106 | -6.50x10^-3 | 0.764 |
| Baseline household income | 8.58x10^-3 | 0.075 | 0.0194 | 0.015* | 0.0305 | 0.05 | 0.0208 | 0.121 | 3.39x10^-3 | 0.695 | 0.0207 | 0.004* | 0.0117 | 0.544 | 0.0205 | 0.326 | 0.0335 | 0.172 |
| Baseline work from home - hybrid | 2.04 | 0.000* | 2.6 | 0.000* | 2.19 | 0.001* | 1.77 | 0.006* | 2.66 | 0.000* | 2.27 | 0.000* | 5.44 | 0.000* | 0.572 | 0.547 | 2.45 | 0.056 |
| Baseline work from home - always | 0.765 | 0.052 | 0.712 | 0.194 | 0.622 | 0.507 | 0.949 | 0.327 | 0.509 | 0.57 | 0.563 | 0.322 | 2.76 | 0.149 | -5.95 | 0.000* | 0.165 | 0.954 |
| Location - North East | 0.873 | 0.188 | 1.72 | 0.068 | 1.32 | 0.556 | -2.11 | 0.14 | -2.26 | 0.053 | 0.681 | 0.485 | -1.43 | 0.781 | 1.96 | 0.498 | -1.37 | 0.646 |
| Location - North West | 0.0907 | 0.854 | 1.42 | 0.061 | 1.85 | 0.157 | -1.25 | 0.235 | 1.24 | 0.163 | 0.0557 | 0.941 | 0.117 | 0.958 | 2.22 | 0.255 | -1.52 | 0.572 |
| Location - Yorkshire | -0.115 | 0.817 | 1.05 | 0.175 | 2.04 | 0.113 | -0.209 | 0.858 | -0.209 | 0.823 | 0.317 | 0.687 | 0.852 | 0.735 | 1.17 | 0.533 | 3.2 | 0.163 |
| Location - East Midlands | 0.208 | 0.679 | 1.19 | 0.125 | 0.739 | 0.553 | 0.0162 | 0.989 | 0.485 | 0.598 | 0.9 | 0.253 | -0.0164 | 0.994 | -0.213 | 0.908 | -2.51 | 0.309 |
| Location - West Midlands | 0.548 | 0.247 | 1.07 | 0.138 | 0.793 | 0.537 | -0.43 | 0.695 | 0.762 | 0.396 | -0.338 | 0.643 | 2.23 | 0.362 | 3.92 | 0.042* | 0.344 | 0.878 |
| Location - East England | 0.176 | 0.714 | 1.48 | 0.048* | -0.0404 | 0.973 | -0.325 | 0.755 | 1.73 | 0.048* | -0.361 | 0.622 | 0.312 | 0.879 | 2.15 | 0.216 | 2.36 | 0.237 |
| Location - South East | -7.96x10^-3 | 0.986 | 1.06 | 0.12 | 0.398 | 0.712 | -1.26 | 0.191 | 0.521 | 0.499 | 0.729 | 0.281 | 0.89 | 0.661 | 0.153 | 0.927 | 0.129 | 0.947 |
| Location - South West | 0.161 | 0.734 | 0.88 | 0.224 | -0.618 | 0.592 | -1.24 | 0.27 | 0.258 | 0.765 | 0.149 | 0.836 | 0.0806 | 0.971 | 1.71 | 0.341 | -0.91 | 0.671 |
| Location - Wales | 0.524 | 0.377 | 2.16 | 0.019* | 0.15 | 0.922 | 0.0882 | 0.946 | 0.141 | 0.893 | 1.18 | 0.201 | 3.03 | 0.193 | 2.55 | 0.242 | 0.917 | 0.701 |
| Location - Scotland | 0.417 | 0.388 | 1.96 | 0.008* | 4.42 | 0.001* | -1.42 | 0.223 | 1.52 | 0.072 | 2.19 | 0.003* | 8.24 | 0.000* | 1.72 | 0.34 | -2.37 | 0.282 |
| Location - Northern Ireland | 0.387 | 0.566 | 0.749 | 0.493 | 2.74 | 0.235 | 0.061 | 0.973 | 0.448 | 0.732 | 0.417 | 0.724 | 0.464 | 0.878 | 1.46 | 0.566 | -0.402 | 0.901 |
| Number of comorbidities | -0.513 | 0.000* | -0.515 | 0.000* | -0.29 | 0.040* | -0.129 | 0.324 | -0.485 | 0.000* | -0.523 | 0.000* | -0.498 | 0.027* | -0.03 | 0.873 | -1.54 | 0.000* |
| Constant | 1.73 | 0.274 | 3.78 | 0.2 | -3.24 | 0.584 | 2.47 | 0.682 | 0.909 | 0.76 | 2.73 | 0.402 | -6.23 | 0.56 | -19.5 | 0.007* | -18.9 | 0.007* |
| N respondents | 10208 |  | 4608 |  | 1659 |  | 1812 |  | 3034 |  | 4482 |  | 796 |  | 1004 |  | 650 |  |
| N observations | 61865 |  | 29463 |  | 10851 |  | 10894 |  | 18841 |  | 28343 |  | 5108 |  | 6195 |  | 3867 |  |
| *Note.* LTC=Long-term condition; *t*=months after April 2020; ENP=emotional, nervous, or psychiatric problem; Coeff.=coefficient; *=significant at 5% level | | | | | | | | | | | | | | | | | | |
