# Supplementary material for: Long-term health conditions and UK labour market outcomes during the COVID-19 pandemic
Source: PLoS One. 2024 May 10;19(5):e0302746. doi: 10.1371/journal.pone.0302746 (PMC11086911; doi:10.1371/journal.pone.0302746)
Supplement: S13 Table — (DOCX) [file pone.0302746.s014.docx]

**Table S13. COVID-19 analysis furlough results table.**

|  | Asthma | | Arthritis | | Cancer | | Diabetes | | ENP | | Vascular | | Pulmonary | | Liver | | Epilepsy | |
| --- | --- | --- | --- | --- | --- | --- | --- | --- | --- | --- | --- | --- | --- | --- | --- | --- | --- | --- |
|  | Coeff. | *p* | Coeff. | *p* | Coeff. | *p* | Coeff. | *p* | Coeff. | *p* | Coeff. | *p* | Coeff. | *p* | Coeff. | *p* | Coeff. | *p* |
| LTC | -0.0309 | 0.596 | 5.98x10^-3 | 0.939 | 0.152 | 0.267 | 0.0475 | 0.716 | -0.13 | 0.168 | 0.0792 | 0.316 | 0.542 | 0.006* | 0.254 | 0.184 | 0.199 | 0.427 |
| ln age | -0.105 | 0.224 | -0.058 | 0.703 | -0.275 | 0.427 | -0.0586 | 0.858 | -0.0723 | 0.648 | -0.0933 | 0.592 | -1.29 | 0.007* | -0.263 | 0.478 | -0.0997 | 0.795 |
| Female | 0.146 | 0.011* | 0.057 | 0.502 | 0.139 | 0.356 | 0.334 | 0.016* | 0.177 | 0.106 | 0.216 | 0.011* | 0.143 | 0.465 | -0.117 | 0.533 | 0.0937 | 0.68 |
| White | 0.316 | 0.001* | 0.258 | 0.067 | 0.105 | 0.73 | 0.721 | 0.000* | 0.134 | 0.496 | 0.281 | 0.047* | 0.752 | 0.112 | 0.658 | 0.039* | 0.225 | 0.591 |
| Household size | 0.0501 | 0.042* | 0.0382 | 0.318 | 0.114 | 0.108 | 0.0901 | 0.15 | 0.0258 | 0.578 | 0.0523 | 0.18 | -3.22x10^-3 | 0.973 | -0.0356 | 0.693 | -9.59x10^-3 | 0.933 |
| Baseline hours worked | 0.0123 | 0.000* | 0.0102 | 0.002* | 9.96x10^-3 | 0.113 | 0.021 | 0.001* | 2.84x10^-3 | 0.496 | 9.03x10^-3 | 0.005* | -3.45x10^-3 | 0.685 | 0.0165 | 0.071 | -1.25x10^-3 | 0.896 |
| Baseline earnings | -0.0221 | 0.000* | -0.0175 | 0.000* | -0.0111 | 0.175 | -0.0122 | 0.088 | -8.71x10^-3 | 0.094 | -0.0116 | 0.004* | -2.11x10^-3 | 0.705 | -5.00x10^-3 | 0.689 | 2.76x10^-3 | 0.597 |
| Baseline household income | -1.30x10^-3 | 0.336 | -1.75x10^-3 | 0.413 | -7.10x10^-3 | 0.11 | -7.16x10^-3 | 0.078 | -4.99x10^-3 | 0.043* | -4.33x10^-3 | 0.061 | -9.47x10^-4 | 0.834 | -0.0143 | 0.020* | -6.75x10^-3 | 0.269 |
| Baseline work from home - hybrid | -0.721 | 0.000* | -0.804 | 0.000* | -1.07 | 0.000* | -0.648 | 0.000* | -0.743 | 0.000* | -0.858 | 0.000* | -1.21 | 0.000* | -0.944 | 0.000* | -1.47 | 0.000* |
| Baseline work from home - always | -1.29 | 0.000* | -1.28 | 0.000* | -1.73 | 0.000* | -1.17 | 0.000* | -1.38 | 0.000* | -1.48 | 0.000* | -0.97 | 0.009* | -1.37 | 0.001* | -1.1 | 0.102 |
| Location - North East | 0.17 | 0.312 | 0.146 | 0.549 | -0.0658 | 0.894 | 0.344 | 0.366 | 0.822 | 0.004* | 0.383 | 0.123 | 1.19 | 0.081 | -0.396 | 0.465 | 0.475 | 0.548 |
| Location - North West | 0.0857 | 0.477 | -0.144 | 0.432 | 0.301 | 0.324 | -0.169 | 0.54 | -0.0404 | 0.859 | -0.0825 | 0.658 | -0.0116 | 0.979 | -0.869 | 0.026* | 0.173 | 0.765 |
| Location - Yorkshire | 0.118 | 0.33 | 0.0239 | 0.896 | 0.121 | 0.711 | -0.309 | 0.264 | 0.0475 | 0.836 | 0.227 | 0.215 | 0.23 | 0.608 | -0.753 | 0.063 | 0.238 | 0.624 |
| Location - East Midlands | 0.26 | 0.035* | 0.0635 | 0.735 | 0.0898 | 0.776 | -0.141 | 0.627 | 0.25 | 0.28 | 0.153 | 0.416 | -0.0197 | 0.964 | -0.247 | 0.552 | 0.891 | 0.082 |
| Location - West Midlands | 0.099 | 0.416 | -0.0206 | 0.91 | 0.126 | 0.697 | 0.0984 | 0.706 | 0.291 | 0.207 | 0.148 | 0.413 | -0.208 | 0.679 | -0.516 | 0.185 | -0.247 | 0.624 |
| Location - East England | 0.0957 | 0.424 | -0.103 | 0.567 | -0.0589 | 0.85 | 6.74x10^-3 | 0.98 | 0.0341 | 0.879 | 0.114 | 0.523 | -0.184 | 0.666 | -0.489 | 0.197 | 0.183 | 0.694 |
| Location - South East | 0.0182 | 0.871 | -0.072 | 0.667 | 0.0186 | 0.947 | -0.492 | 0.052 | 0.108 | 0.601 | -0.173 | 0.31 | 0.149 | 0.713 | -0.592 | 0.095 | 0.297 | 0.488 |
| Location - South West | -0.0188 | 0.879 | 3.03x10^-3 | 0.987 | 0.258 | 0.383 | -0.193 | 0.486 | 0.214 | 0.334 | 0.193 | 0.279 | -0.199 | 0.644 | -0.355 | 0.361 | 0.292 | 0.538 |
| Location - Wales | 0.063 | 0.646 | -0.195 | 0.347 | 0.424 | 0.209 | -0.0313 | 0.917 | 0.196 | 0.429 | 0.0346 | 0.867 | -0.526 | 0.266 | -0.707 | 0.119 | 0.525 | 0.341 |
| Location - Scotland | -0.0996 | 0.435 | -0.17 | 0.375 | -0.421 | 0.244 | -0.757 | 0.019* | 0.0456 | 0.844 | -0.115 | 0.546 | -0.758 | 0.116 | -0.718 | 0.097 | -0.0346 | 0.947 |
| Location - Northern Ireland | 0.0422 | 0.786 | 0.125 | 0.592 | -0.0177 | 0.968 | -0.314 | 0.458 | 0.369 | 0.206 | 0.202 | 0.41 | -0.0698 | 0.909 | -0.928 | 0.106 | 0.554 | 0.409 |
| Number of comorbidities | 0.0555 | 0.011* | 0.047 | 0.07 | 0.0412 | 0.227 | -5.89x10^-3 | 0.862 | 0.0571 | 0.044* | 0.0328 | 0.174 | 0.0391 | 0.368 | 3.83x10^-3 | 0.934 | 0.0543 | 0.304 |
| Constant | 0.0796 | 0.832 | 0.13 | 0.847 | 0.761 | 0.624 | -0.394 | 0.78 | 0.241 | 0.726 | 0.0559 | 0.942 | 5.09 | 0.015* | 1.4 | 0.379 | 0.448 | 0.793 |
| N | 10208 |  | 4608 |  | 1659 |  | 1812 |  | 3034 |  | 4482 |  | 796 |  | 1004 |  | 650 |  |
| *Note.* LTC=Long-term condition; ENP=emotional, nervous, or psychiatric problem; Coeff.=coefficient; *=significant at 5% level | | | | | | | | | | | | | | | | | | |
