# Supplementary material for: Long-term health conditions and UK labour market outcomes during the COVID-19 pandemic
Source: PLoS One. 2024 May 10;19(5):e0302746. doi: 10.1371/journal.pone.0302746 (PMC11086911; doi:10.1371/journal.pone.0302746)
Supplement: S14 Table — (DOCX) [file pone.0302746.s015.docx]

**Table S14. COVID-19 analysis working from home results.**

|  | Asthma | | Arthritis | | Cancer | | Diabetes | | ENP | | Vascular | | Pulmonary | | Liver | | Epilepsy | |
| --- | --- | --- | --- | --- | --- | --- | --- | --- | --- | --- | --- | --- | --- | --- | --- | --- | --- | --- |
|  | Coeff. | *p* | Coeff. | *p* | Coeff. | *p* | Coeff. | *p* | Coeff. | *p* | Coeff. | *p* | Coeff. | *p* | Coeff. | *p* | Coeff. | *p* |
| LTC | -0.0105 | 0.913 | -0.209 | 0.083 | 0.0986 | 0.62 | 0.0226 | 0.912 | 0.145 | 0.275 | -4.58x10^-4 | 0.997 | 0.216 | 0.509 | -0.0952 | 0.734 | 0.139 | 0.722 |
| *t* | -0.0581 | 0.000* | -0.0624 | 0.000* | -0.0592 | 0.000* | -0.0573 | 0.000* | -0.0588 | 0.000* | -0.0596 | 0.000* | -0.0583 | 0.000* | -0.0582 | 0.000* | -0.0577 | 0.000* |
| LTC × *t* | 6.32x10^-4 | 0.917 | 0.0251 | 0.001* | 0.031 | 0.042* | -0.0133 | 0.34 | 7.58x10^-3 | 0.419 | 9.47x10^-3 | 0.213 | 0.035 | 0.173 | 0.0158 | 0.453 | -0.0143 | 0.661 |
| ln age | -1.19 | 0.000* | -1.18 | 0.000* | -1.21 | 0.000* | -1.19 | 0.000* | -1.16 | 0.000* | -1.19 | 0.000* | -1.2 | 0.000* | -1.19 | 0.000* | -1.19 | 0.000* |
| Female | 0.469 | 0.000* | 0.472 | 0.000* | 0.465 | 0.000* | 0.467 | 0.000* | 0.46 | 0.000* | 0.471 | 0.000* | 0.47 | 0.000* | 0.468 | 0.000* | 0.469 | 0.000* |
| White | -0.158 | 0.196 | -0.159 | 0.192 | -0.162 | 0.184 | -0.16 | 0.189 | -0.166 | 0.173 | -0.157 | 0.196 | -0.159 | 0.192 | -0.158 | 0.196 | -0.158 | 0.195 |
| Household size | -0.0529 | 0.028* | -0.0526 | 0.029* | -0.0524 | 0.029* | -0.0526 | 0.029* | -0.0525 | 0.029* | -0.0526 | 0.029* | -0.0531 | 0.027* | -0.053 | 0.027* | -0.0529 | 0.028* |
| Baseline hours worked | 0.0121 | 0.000* | 0.0121 | 0.000* | 0.0122 | 0.000* | 0.0121 | 0.000* | 0.0121 | 0.000* | 0.0121 | 0.000* | 0.012 | 0.000* | 0.0121 | 0.000* | 0.0121 | 0.000* |
| Baseline earnings | 0.0257 | 0.000* | 0.0258 | 0.000* | 0.0258 | 0.000* | 0.0258 | 0.000* | 0.0258 | 0.000* | 0.0258 | 0.000* | 0.0258 | 0.000* | 0.0258 | 0.000* | 0.0258 | 0.000* |
| Baseline household income | 1.05x10^-3 | 0.99 | 1.05x10^-3 | 0.99 | 1.04x10^-3 | 0.99 | 1.04x10^-3 | 0.99 | 1.06x10^-3 | 0.99 | 1.05x10^-3 | 0.99 | 1.04x10^-3 | 0.99 | 1.04x10^-3 | 0.99 | 1.04x10^-3 | 0.99 |
| Baseline work from home - hybrid | 3.41 | 0.000* | 3.41 | 0.000* | 3.41 | 0.000* | 3.41 | 0.000* | 3.41 | 0.000* | 3.41 | 0.000* | 3.41 | 0.000* | 3.41 | 0.000* | 3.41 | 0.000* |
| Baseline work from home - always | 5.12 | 0.000* | 5.12 | 0.000* | 5.12 | 0.000* | 5.12 | 0.000* | 5.12 | 0.000* | 5.12 | 0.000* | 5.12 | 0.000* | 5.12 | 0.000* | 5.12 | 0.000* |
| Location - North East | -1.21 | 0.000* | -1.22 | 0.000* | -1.21 | 0.000* | -1.21 | 0.000* | -1.22 | 0.000* | -1.21 | 0.000* | -1.21 | 0.000* | -1.21 | 0.000* | -1.21 | 0.000* |
| Location - North West | 3.76 | 0.000* | 3.75 | 0.000* | 3.75 | 0.000* | 3.75 | 0.000* | 3.75 | 0.000* | 3.75 | 0.000* | 3.76 | 0.000* | 3.75 | 0.000* | 3.76 | 0.000* |
| Location - Yorkshire | 2.48 | 0.000* | 2.48 | 0.000* | 2.48 | 0.000* | 2.48 | 0.000* | 2.48 | 0.000* | 2.48 | 0.000* | 2.48 | 0.000* | 2.48 | 0.000* | 2.48 | 0.000* |
| Location - East Midlands | -0.643 | 0.000* | -0.638 | 0.000* | -0.641 | 0.000* | -0.646 | 0.000* | -0.647 | 0.000* | -0.643 | 0.000* | -0.643 | 0.000* | -0.644 | 0.000* | -0.644 | 0.000* |
| Location - West Midlands | -0.532 | 0.002* | -0.53 | 0.002* | -0.533 | 0.002* | -0.534 | 0.002* | -0.532 | 0.002* | -0.532 | 0.002* | -0.536 | 0.002* | -0.533 | 0.002* | -0.532 | 0.002* |
| Location - East England | -0.752 | 0.000* | -0.753 | 0.000* | -0.754 | 0.000* | -0.755 | 0.000* | -0.753 | 0.000* | -0.753 | 0.000* | -0.755 | 0.000* | -0.754 | 0.000* | -0.753 | 0.000* |
| Location - South East | -0.81 | 0.000* | -0.802 | 0.000* | -0.813 | 0.000* | -0.812 | 0.000* | -0.809 | 0.000* | -0.811 | 0.000* | -0.813 | 0.000* | -0.811 | 0.000* | -0.811 | 0.000* |
| Location - South West | -0.62 | 0.000* | -0.622 | 0.000* | -0.621 | 0.000* | -0.621 | 0.000* | -0.62 | 0.000* | -0.622 | 0.000* | -0.62 | 0.000* | -0.621 | 0.000* | -0.62 | 0.000* |
| Location - Wales | -0.49 | 0.001* | -0.489 | 0.001* | -0.488 | 0.001* | -0.491 | 0.001* | -0.491 | 0.001* | -0.489 | 0.001* | -0.491 | 0.001* | -0.49 | 0.001* | -0.49 | 0.001* |
| Location - Scotland | -0.6 | 0.000* | -0.598 | 0.000* | -0.602 | 0.000* | -0.601 | 0.000* | -0.601 | 0.000* | -0.6 | 0.000* | -0.603 | 0.000* | -0.601 | 0.000* | -0.601 | 0.000* |
| Location - Northern Ireland | -0.551 | 0.004* | -0.55 | 0.005* | -0.556 | 0.004* | -0.553 | 0.004* | -0.552 | 0.004* | -0.551 | 0.004* | -0.556 | 0.004* | -0.552 | 0.004* | -0.552 | 0.004* |
| Number of comorbidities | -0.944 | 0.000* | -0.942 | 0.000* | -0.944 | 0.000* | -0.946 | 0.000* | -0.94 | 0.000* | -0.945 | 0.000* | -0.947 | 0.000* | -0.945 | 0.000* | -0.945 | 0.000* |
| σ | -0.282 | 0.173 | -0.282 | 0.173 | -0.279 | 0.178 | -0.284 | 0.17 | -0.285 | 0.169 | -0.283 | 0.171 | -0.284 | 0.17 | -0.284 | 0.17 | -0.283 | 0.172 |
| τ_1_ | -0.503 | 0.000* | -0.505 | 0.000* | -0.504 | 0.000* | -0.504 | 0.000* | -0.504 | 0.000* | -0.504 | 0.000* | -0.505 | 0.000* | -0.503 | 0.000* | -0.503 | 0.000* |
| τ_2_ | 0.025 | 0.481 | 0.0369 | 0.254 | 0.0105 | 0.68 | 0.0207 | 0.428 | -1.68x10^-3 | 0.953 | 0.0124 | 0.679 | 0.0106 | 0.674 | 0.0181 | 0.476 | 0.017 | 0.493 |
| N respondents | 3.33 | 0.000* | 3.33 | 0.000* | 3.33 | 0.000* | 3.33 | 0.000* | 3.33 | 0.000* | 3.33 | 0.000* | 3.33 | 0.000* | 3.33 | 0.000* | 3.33 | 0.000* |
| N observations | -1.28 | 0.015* | -1.23 | 0.019* | -1.32 | 0.012* | -1.27 | 0.015* | -1.18 | 0.026* | -1.28 | 0.015* | -1.28 | 0.014* | -1.27 | 0.015* | -1.26 | 0.016* |
| *Note.* LTC=Long-term condition; *t*=months after April 2020; ENP=emotional, nervous, or psychiatric problem; Coeff.=coefficient; *=significant at 5% level | | | | | | | | | | | | | | | | | | |
