# Supplementary material for: Long-term health conditions and UK labour market outcomes during the COVID-19 pandemic
Source: PLoS One. 2024 May 10;19(5):e0302746. doi: 10.1371/journal.pone.0302746 (PMC11086911; doi:10.1371/journal.pone.0302746)
Supplement: S15 Table — (DOCX) [file pone.0302746.s016.docx]

**Table S15. COVID-19 analysis earnings conditional on employment results.**

|  | Asthma | | Arthritis | | Cancer | | Diabetes | | ENP | | Vascular | | Pulmonary | | Liver | | Epilepsy | |
| --- | --- | --- | --- | --- | --- | --- | --- | --- | --- | --- | --- | --- | --- | --- | --- | --- | --- | --- |
|  | Coeff. | *p* | Coeff. | *p* | Coeff. | *p* | Coeff. | *p* | Coeff. | *p* | Coeff. | *p* | Coeff. | *p* | Coeff. | *p* | Coeff. | *p* |
| LTC | -0.224 | 0.241 | -0.244 | 0.457 | 0.265 | 0.561 | -1.16 | 0.105 | -0.744 | 0.067 | 0.238 | 0.496 | 0.376 | 0.756 | -0.696 | 0.434 | -1.64 | 0.119 |
| *t* | 0.124 | 0.000* | 0.0799 | 0.000* | 0.0961 | 0.000* | 0.0859 | 0.013* | 0.0727 | 0.021* | 0.133 | 0.000* | 0.131 | 0.035* | 0.223 | 0.000* | 0.115 | 0.020* |
| LTC × *t* | -0.0204 | 0.255 | 0.0612 | 0.018* | 0.0709 | 0.040* | 0.0491 | 0.433 | 0.067 | 0.084 | -0.0155 | 0.577 | -0.0652 | 0.523 | -0.0172 | 0.85 | -0.0595 | 0.437 |
| ln age | 0.887 | 0.008* | 0.16 | 0.774 | 3.98 | 0.000* | -1.28 | 0.45 | 0.988 | 0.183 | 1.32 | 0.064 | -2.02 | 0.375 | 0.0244 | 0.988 | 2.39 | 0.141 |
| Female | -2.78 | 0.000* | -2.56 | 0.000* | -1.36 | 0.000* | -1.34 | 0.046* | -2.6 | 0.000* | -2.81 | 0.000* | -2.71 | 0.008* | 0.485 | 0.542 | -2.12 | 0.012* |
| White | 1.49 | 0.000* | 1.13 | 0.005* | -0.11 | 0.883 | 1.91 | 0.010* | 2.09 | 0.000* | 1.35 | 0.005* | -1.61 | 0.372 | -1.03 | 0.273 | -8.42 | 0.000* |
| Household size | 0.0407 | 0.479 | 0.178 | 0.043* | -0.0382 | 0.781 | -0.164 | 0.478 | -0.221 | 0.116 | -0.19 | 0.076 | -0.0696 | 0.843 | -0.148 | 0.648 | -0.208 | 0.578 |
| Baseline hours worked | 0.161 | 0.000* | 0.172 | 0.000* | 0.137 | 0.000* | 0.124 | 0.000* | 0.131 | 0.000* | 0.13 | 0.000* | 0.207 | 0.000* | 0.0122 | 0.676 | 0.329 | 0.000* |
| Baseline earnings | 0.446 | 0.000* | 0.372 | 0.000* | 0.555 | 0.000* | 0.491 | 0.000* | 0.412 | 0.000* | 0.409 | 0.000* | 0.243 | 0.000* | 0.903 | 0.000* | 0.256 | 0.000* |
| Baseline household income | 0.0839 | 0.000* | 0.0849 | 0.000* | 0.17 | 0.000* | 0.105 | 0.000* | 0.0741 | 0.000* | 0.178 | 0.000* | 0.0846 | 0.000* | -5.06x10^-3 | 0.808 | 0.193 | 0.000* |
| Baseline work from home - hybrid | 2.51 | 0.000* | 2.43 | 0.000* | 1.99 | 0.000* | 1.18 | 0.095 | 1.75 | 0.000* | 2.83 | 0.000* | 4.23 | 0.000* | 1.95 | 0.004* | 4.32 | 0.000* |
| Baseline work from home - always | -0.706 | 0.018* | -2.43 | 0.000* | -0.515 | 0.526 | 1.25 | 0.266 | -2.06 | 0.023* | 0.165 | 0.747 | 0.101 | 0.95 | -2.94 | 0.028* | 0.584 | 0.776 |
| Location - North East | -1.71 | 0.000* | -0.354 | 0.753 | -3.65 | 0.123 | -2.6 | 0.242 | -1.07 | 0.401 | -1.29 | 0.074 | -4.44 | 0.542 | -0.0691 | 0.975 | -5.41 | 0.126 |
| Location - North West | -1.81 | 0.000* | 0.151 | 0.796 | -4.12 | 0.000* | -3.09 | 0.025* | -0.016 | 0.983 | -0.6 | 0.312 | -2.02 | 0.242 | -2.19 | 0.167 | -1.33 | 0.554 |
| Location - Yorkshire | -2.16 | 0.000* | -0.497 | 0.472 | -5 | 0.000* | -2.84 | 0.032* | -0.378 | 0.692 | -2.72 | 0.000* | -3.6 | 0.152 | -2.11 | 0.358 | -2.64 | 0.223 |
| Location - East Midlands | -1.85 | 0.000* | -1.42 | 0.05 | -5.2 | 0.000* | -3.08 | 0.040* | -1.33 | 0.212 | -1.35 | 0.142 | -5.03 | 0.023* | -1.61 | 0.25 | -0.749 | 0.728 |
| Location - West Midlands | -0.567 | 0.132 | 0.497 | 0.318 | -4.06 | 0.000* | -2.76 | 0.024* | 0.39 | 0.624 | -0.0742 | 0.904 | -4.33 | 0.062 | 0.26 | 0.825 | -0.738 | 0.664 |
| Location - East England | -0.38 | 0.139 | 0.033 | 0.942 | -6.04 | 0.000* | 0.351 | 0.719 | 1.08 | 0.051 | -1.91 | 0.000* | -2.36 | 0.101 | -1.05 | 0.355 | -0.997 | 0.487 |
| Location - South East | -0.594 | 0.026* | 0.319 | 0.439 | -2.74 | 0.000* | -1.55 | 0.101 | -0.176 | 0.757 | -0.16 | 0.721 | -3.72 | 0.036* | -1.56 | 0.212 | 1.47 | 0.27 |
| Location - South West | -1.3 | 0.000* | -0.368 | 0.469 | -3.42 | 0.000* | -3.29 | 0.003* | -0.144 | 0.843 | -1.16 | 0.045* | -4.05 | 0.007* | -1.35 | 0.245 | 0.211 | 0.881 |
| Location - Wales | -0.451 | 0.246 | 1.01 | 0.121 | -5.5 | 0.000* | -1.22 | 0.47 | 0.85 | 0.339 | 1.09 | 0.102 | -2.28 | 0.253 | 1.47 | 0.299 | -1.01 | 0.623 |
| Location - Scotland | -1.69 | 0.000* | -0.591 | 0.314 | -1.78 | 0.077 | -1.98 | 0.251 | 0.136 | 0.89 | 0.15 | 0.827 | -0.227 | 0.916 | -1.9 | 0.274 | -0.269 | 0.886 |
| Location - Northern Ireland | -1.97 | 0.000* | 0.902 | 0.256 | -5.96 | 0.000* | -0.261 | 0.862 | -0.927 | 0.552 | 0.517 | 0.58 | 2.37 | 0.244 | -0.849 | 0.645 | -5.03 | 0.011* |
| Number of comorbidities | -0.086 | 0.17 | -0.0283 | 0.728 | 0.594 | 0.000* | -0.153 | 0.367 | -0.289 | 0.002* | -0.276 | 0.001* | -0.0597 | 0.776 | -0.0106 | 0.942 | 0.135 | 0.457 |
| Constant | -2.96 | 0.033* | -1.01 | 0.671 | -18.1 | 0.000* | 6.55 | 0.366 | -1.46 | 0.631 | -5.6 | 0.064 | 14.4 | 0.154 | 1.88 | 0.79 | -5.2 | 0.431 |
| N respondents | 10208 |  | 4608 |  | 1659 |  | 1812 |  | 3034 |  | 4482 |  | 796 |  | 1004 |  | 650 |  |
| N observations | 61865 |  | 29463 |  | 10851 |  | 10894 |  | 18841 |  | 28343 |  | 5108 |  | 6195 |  | 3867 |  |
| *Note.* LTC=Long-term condition; *t*=months after April 2020; ENP=emotional, nervous, or psychiatric problem; Coeff.=coefficient; *=significant at 5% level | | | | | | | | | | | | | | | | | | |
