# Supplementary material for: Long-term health conditions and UK labour market outcomes during the COVID-19 pandemic
Source: PLoS One. 2024 May 10;19(5):e0302746. doi: 10.1371/journal.pone.0302746 (PMC11086911; doi:10.1371/journal.pone.0302746)
Supplement: S16 Table — (DOCX) [file pone.0302746.s017.docx]

**Table S16. Asthma Mahalanobis distance matching for pre-COVID-19 data.**

|  |  | Treatment | | Control | | SMD |
| --- | --- | --- | --- | --- | --- | --- |
|  |  | N | % | N | % |  |
| Age | mean (sd) | 46.4 | 12.6 | 42.5 | 12.1 | 0.309 |
| Female |  | 4880 | 54.2 | 4842 | 53.7 | 8.46x10^-3 |
| White |  | 7592 | 84.3 | 7550 | 83.8 | 0.0128 |
| Baseline hours worked | mean (sd) | 36.5 | 17.1 | 37 | 16.3 | -0.0322 |
| Baseline earnings | mean (sd) | 18.2 | 13.6 | 18.8 | 12.1 | -0.0431 |
| Job category | professional | 3715 | 41.2 | 3733 | 41.4 | 7.34x10^-3 |
|  | intermediate | 2264 | 25.1 | 2285 | 25.4 |  |
|  | routine | 3031 | 33.6 | 2992 | 33.2 |  |
| Location | North East | 328 | 3.6 | 264 | 2.9 | -0.0154 |
|  | North West | 907 | 10.1 | 904 | 10 |  |
|  | Yorkshire | 769 | 8.5 | 774 | 8.6 |  |
|  | East Midlands | 699 | 7.8 | 672 | 7.5 |  |
|  | West Midlands | 741 | 8.2 | 723 | 8 |  |
|  | East England | 796 | 8.8 | 761 | 8.4 |  |
|  | South East | 1131 | 12.6 | 1143 | 12.7 |  |
|  | South West | 759 | 8.4 | 729 | 8.1 |  |
|  | London | 1108 | 12.3 | 1290 | 14.3 |  |
|  | Wales | 571 | 6.3 | 571 | 6.3 |  |
|  | Scotland | 763 | 8.5 | 705 | 7.8 |  |
|  | Northern Ireland | 434 | 4.8 | 470 | 5.2 |  |
| Household size | mean (sd) | 3 | 1.4 | 3.1 | 1.4 | -0.0905 |
| Baseline household income | mean (sd) | 47 | 98.1 | 47.3 | 97.1 | -3.72x10^-3 |
| Number of comorbidities | mean (sd) | 1.7 | 1.6 | 0.4 | 0.7 | 0.81 |
| N |  | 9010 |  | 9010 |  |  |
| *Note.* SMD=standardised mean difference | | | | | | |
