# Supplementary material for: Long-term health conditions and UK labour market outcomes during the COVID-19 pandemic
Source: PLoS One. 2024 May 10;19(5):e0302746. doi: 10.1371/journal.pone.0302746 (PMC11086911; doi:10.1371/journal.pone.0302746)
Supplement: S17 Table — (DOCX) [file pone.0302746.s018.docx]

**Table S17. Arthritis Mahalanobis distance matching for pre-COVID-19 data.**

|  |  | Treatment | | Control | | SMD |
| --- | --- | --- | --- | --- | --- | --- |
|  |  | N | % | N | % |  |
| Age | mean (sd) | 50.9 | 11 | 48.5 | 10.9 | 0.22 |
| Female |  | 2477 | 58.7 | 2466 | 58.4 | 5.29x10^-3 |
| White |  | 3639 | 86.2 | 3637 | 86.1 | 1.37x10^-3 |
| Baseline hours worked | mean (sd) | 35.1 | 17 | 35.6 | 16.4 | -0.0274 |
| Baseline earnings | mean (sd) | 17.2 | 11.8 | 17.5 | 11.5 | -0.0328 |
| Job category | professional | 1609 | 38.1 | 1637 | 38.8 | 0.0168 |
|  | intermediate | 1086 | 25.7 | 1091 | 25.8 |  |
|  | routine | 1527 | 36.2 | 1494 | 35.4 |  |
| Location | North East | 175 | 4.1 | 130 | 3.1 | -0.0162 |
|  | North West | 458 | 10.8 | 422 | 10 |  |
|  | Yorkshire | 341 | 8.1 | 376 | 8.9 |  |
|  | East Midlands | 343 | 8.1 | 325 | 7.7 |  |
|  | West Midlands | 345 | 8.2 | 352 | 8.3 |  |
|  | East England | 351 | 8.3 | 382 | 9 |  |
|  | South East | 540 | 12.8 | 540 | 12.8 |  |
|  | South West | 367 | 8.7 | 371 | 8.8 |  |
|  | London | 448 | 10.6 | 506 | 12 |  |
|  | Wales | 287 | 6.8 | 245 | 5.8 |  |
|  | Scotland | 356 | 8.4 | 367 | 8.7 |  |
|  | Northern Ireland | 209 | 5 | 204 | 4.8 |  |
| Household size | mean (sd) | 2.8 | 1.3 | 2.9 | 1.3 | -0.0503 |
| Baseline household income | mean (sd) | 44.2 | 35.3 | 44.7 | 34.5 | -0.0139 |
| Number of comorbidities | mean (sd) | 2.6 | 1.8 | 1.9 | 1.2 | 0.375 |
| N |  | 4222 |  | 4222 |  |  |
| *Note.* SMD=standardised mean difference | | | | | | |
