# Supplementary material for: Long-term health conditions and UK labour market outcomes during the COVID-19 pandemic
Source: PLoS One. 2024 May 10;19(5):e0302746. doi: 10.1371/journal.pone.0302746 (PMC11086911; doi:10.1371/journal.pone.0302746)
Supplement: S18 Table — (DOCX) [file pone.0302746.s019.docx]

**Table S18. Cancer Mahalanobis distance matching for pre-COVID-19 data.**

|  |  | Treatment | | Control | | SMD |
| --- | --- | --- | --- | --- | --- | --- |
|  |  | N | % | N | % |  |
| Age | mean (sd) | 53.2 | 10.4 | 52 | 10.2 | 0.115 |
| Female |  | 635 | 61.5 | 634 | 61.4 | 1.99x10^-3 |
| White |  | 945 | 91.5 | 946 | 91.6 | -3.47x10^-3 |
| Baseline hours worked | mean (sd) | 35.4 | 17.8 | 35.3 | 16.5 | 4.02x10^-3 |
| Baseline earnings | mean (sd) | 18.7 | 13.6 | 18.5 | 13 | 0.0164 |
| Job category | professional | 476 | 46.1 | 480 | 46.5 | 5.77x10^-3 |
|  | intermediate | 272 | 26.3 | 269 | 26 |  |
|  | routine | 285 | 27.6 | 284 | 27.5 |  |
| Location | North East | 25 | 2.4 | 24 | 2.3 | -8.45x10^-3 |
|  | North West | 100 | 9.7 | 96 | 9.3 |  |
|  | Yorkshire | 77 | 7.5 | 82 | 7.9 |  |
|  | East Midlands | 85 | 8.2 | 87 | 8.4 |  |
|  | West Midlands | 84 | 8.1 | 83 | 8 |  |
|  | East England | 89 | 8.6 | 78 | 7.6 |  |
|  | South East | 161 | 15.6 | 153 | 14.8 |  |
|  | South West | 106 | 10.3 | 114 | 11 |  |
|  | London | 111 | 10.7 | 113 | 10.9 |  |
|  | Wales | 50 | 4.8 | 63 | 6.1 |  |
|  | Scotland | 75 | 7.3 | 78 | 7.6 |  |
|  | Northern Ireland | 70 | 6.8 | 61 | 5.9 |  |
| Household size | mean (sd) | 2.8 | 1.3 | 2.8 | 1.3 | -1.20x10^-3 |
| Baseline household income | mean (sd) | 49.9 | 37.8 | 46.4 | 31.7 | 0.0939 |
| Number of comorbidities | mean (sd) | 2.2 | 2.1 | 2 | 2 | 0.0614 |
| N |  | 1033 |  | 1033 |  |  |
| *Note.* SMD=standardised mean difference | | | | | | |
