# Supplementary material for: Long-term health conditions and UK labour market outcomes during the COVID-19 pandemic
Source: PLoS One. 2024 May 10;19(5):e0302746. doi: 10.1371/journal.pone.0302746 (PMC11086911; doi:10.1371/journal.pone.0302746)
Supplement: S19 Table — (DOCX) [file pone.0302746.s020.docx]

**Table S19. Diabetes Mahalanobis score matching for pre-COVID-19 data.**

|  |  | Treatment | | Control | | SMD |
| --- | --- | --- | --- | --- | --- | --- |
|  |  | N | % | N | % |  |
| Age | mean (sd) | 51.1 | 10.8 | 50.2 | 10.2 | 0.0794 |
| Female |  | 562 | 42.4 | 559 | 42.1 | 4.58x10^-3 |
| White |  | 926 | 69.8 | 927 | 69.9 | -1.64x10^-3 |
| Baseline hours worked | mean (sd) | 35.7 | 16.4 | 36 | 15.9 | -0.0193 |
| Baseline earnings | mean (sd) | 17.6 | 12.6 | 17.9 | 12.1 | -0.0207 |
| Job category | professional | 487 | 36.7 | 490 | 36.9 | 0 |
|  | intermediate | 324 | 24.4 | 318 | 24 |  |
|  | routine | 516 | 38.9 | 519 | 39.1 |  |
| Location | North East | 39 | 2.9 | 30 | 2.3 | -0.0148 |
|  | North West | 148 | 11.2 | 152 | 11.5 |  |
|  | Yorkshire | 111 | 8.4 | 99 | 7.5 |  |
|  | East Midlands | 108 | 8.1 | 112 | 8.4 |  |
|  | West Midlands | 124 | 9.3 | 130 | 9.8 |  |
|  | East England | 107 | 8.1 | 98 | 7.4 |  |
|  | South East | 159 | 12 | 147 | 11.1 |  |
|  | South West | 92 | 6.9 | 98 | 7.4 |  |
|  | London | 233 | 17.6 | 256 | 19.3 |  |
|  | Wales | 68 | 5.1 | 77 | 5.8 |  |
|  | Scotland | 85 | 6.4 | 83 | 6.3 |  |
|  | Northern Ireland | 51 | 3.8 | 45 | 3.4 |  |
| Household size | mean (sd) | 3.2 | 1.6 | 3.2 | 1.6 | 5.11x10^-3 |
| Baseline household income | mean (sd) | 57.9 | 336.9 | 50.7 | 240.4 | 0.0212 |
| Number of comorbidities | mean (sd) | 2.8 | 2.1 | 2.6 | 2 | 0.106 |
| N |  | 1327 |  | 1327 |  |  |
| *Note.* SMD=standardised mean difference | | | | | | |
