# Supplementary material for: Long-term health conditions and UK labour market outcomes during the COVID-19 pandemic
Source: PLoS One. 2024 May 10;19(5):e0302746. doi: 10.1371/journal.pone.0302746 (PMC11086911; doi:10.1371/journal.pone.0302746)
Supplement: S20 Table — (DOCX) [file pone.0302746.s021.docx]

**Table S20. Emotional, nervous or psychiatric problem Mahalanobis distance score matching for pre-COVID-19 data.**

|  |  | Treatment | | Control | | SMD |
| --- | --- | --- | --- | --- | --- | --- |
|  |  | N | % | N | % |  |
| Age | mean (sd) | 43.6 | 11.9 | 44 | 12 | -0.0385 |
| Female |  | 1533 | 67.5 | 1527 | 67.2 | 5.64x10^-3 |
| White |  | 2062 | 90.8 | 2061 | 90.8 | 1.52x10^-3 |
| Baseline hours worked | mean (sd) | 35.2 | 16.7 | 35.3 | 15.8 | -7.39x10^-3 |
| Baseline earnings | mean (sd) | 16.6 | 11.1 | 17 | 10.7 | -0.0339 |
| Job category | professional | 972 | 42.8 | 974 | 42.9 | -3.56x10^-3 |
|  | intermediate | 544 | 24 | 533 | 23.5 |  |
|  | routine | 755 | 33.2 | 764 | 33.6 |  |
| Location | North East | 88 | 3.9 | 60 | 2.6 | -0.0168 |
|  | North West | 232 | 10.2 | 246 | 10.8 |  |
|  | Yorkshire | 193 | 8.5 | 195 | 8.6 |  |
|  | East Midlands | 162 | 7.1 | 187 | 8.2 |  |
|  | West Midlands | 166 | 7.3 | 152 | 6.7 |  |
|  | East England | 202 | 8.9 | 179 | 7.9 |  |
|  | South East | 333 | 14.7 | 313 | 13.8 |  |
|  | South West | 217 | 9.6 | 198 | 8.7 |  |
|  | London | 226 | 10 | 262 | 11.5 |  |
|  | Wales | 151 | 6.6 | 169 | 7.4 |  |
|  | Scotland | 214 | 9.4 | 202 | 8.9 |  |
|  | Northern Ireland | 87 | 3.8 | 108 | 4.8 |  |
| Household size | mean (sd) | 2.9 | 1.3 | 2.8 | 1.3 | 1.27x10^-3 |
| Baseline household income | mean (sd) | 45.7 | 183.3 | 45.4 | 182.8 | 1.52x10^-3 |
| Number of comorbidities | mean (sd) | 2.4 | 1.9 | 2.2 | 1.8 | 0.101 |
| N |  | 2271 |  | 2271 |  |  |
| *Note.* SMD=standardised mean difference | | | | | | |
