# Supplementary material for: Long-term health conditions and UK labour market outcomes during the COVID-19 pandemic
Source: PLoS One. 2024 May 10;19(5):e0302746. doi: 10.1371/journal.pone.0302746 (PMC11086911; doi:10.1371/journal.pone.0302746)
Supplement: S21 Table — (DOCX) [file pone.0302746.s022.docx]

**Table S21. Vascular conditions Mahalanobis distance matching for pre-COVID-19 data.**

|  |  | Treatment | | Control | | SMD |
| --- | --- | --- | --- | --- | --- | --- |
|  |  | N | % | N | % |  |
| Age | mean (sd) | 51.4 | 10.8 | 48.8 | 10.5 | 0.241 |
| Female |  | 2085 | 47.1 | 2095 | 47.3 | -4.53x10^-3 |
| White |  | 3676 | 83.1 | 3670 | 82.9 | 3.61x10^-3 |
| Baseline hours worked | mean (sd) | 36.8 | 17.2 | 36.7 | 16.3 | 5.04x10^-3 |
| Baseline earnings | mean (sd) | 18.6 | 15.1 | 18.8 | 12.5 | -0.0165 |
| Job category | professional | 1777 | 40.1 | 1790 | 40.4 | 7.33x10^-3 |
|  | intermediate | 1119 | 25.3 | 1121 | 25.3 |  |
|  | routine | 1530 | 34.6 | 1515 | 34.2 |  |
| Location | North East | 142 | 3.2 | 139 | 3.1 | -3.13x10^-3 |
|  | North West | 445 | 10.1 | 412 | 9.3 |  |
|  | Yorkshire | 350 | 7.9 | 356 | 8 |  |
|  | East Midlands | 346 | 7.8 | 370 | 8.4 |  |
|  | West Midlands | 363 | 8.2 | 385 | 8.7 |  |
|  | East England | 383 | 8.7 | 357 | 8.1 |  |
|  | South East | 579 | 13.1 | 583 | 13.2 |  |
|  | South West | 364 | 8.2 | 397 | 9 |  |
|  | London | 599 | 13.5 | 597 | 13.5 |  |
|  | Wales | 274 | 6.2 | 261 | 5.9 |  |
|  | Scotland | 359 | 8.1 | 367 | 8.3 |  |
|  | Northern Ireland | 219 | 4.9 | 201 | 4.5 |  |
| Household size | mean (sd) | 2.9 | 1.4 | 3 | 1.3 | -0.0456 |
| Baseline household income | mean (sd) | 45.8 | 37.1 | 45.2 | 32.9 | 0.018 |
| Number of comorbidities | mean (sd) | 2.3 | 1.9 | 1.7 | 1.3 | 0.306 |
| N |  | 4426 |  | 4426 |  |  |
| *Note.* SMD=standardised mean difference | | | | | | |
