# Supplementary material for: Long-term health conditions and UK labour market outcomes during the COVID-19 pandemic
Source: PLoS One. 2024 May 10;19(5):e0302746. doi: 10.1371/journal.pone.0302746 (PMC11086911; doi:10.1371/journal.pone.0302746)
Supplement: S22 Table — (DOCX) [file pone.0302746.s023.docx]

**Table S22. Pulmonary conditions Mahalanobis distance matching for pre-COVID-19 data.**

|  |  | Treatment | | Control | | SMD |
| --- | --- | --- | --- | --- | --- | --- |
|  |  | N | % | N | % |  |
| Age | mean (sd) | 53 | 10.8 | 51.7 | 10.4 | 0.128 |
| Female |  | 231 | 53.2 | 691 | 53.1 | 3.08x10^-3 |
| White |  | 411 | 94.7 | 1232 | 94.6 | 3.43x10^-3 |
| Baseline hours worked | mean (sd) | 36.2 | 15.5 | 35.7 | 14.7 | 0.0316 |
| Baseline earnings | mean (sd) | 17.2 | 12.6 | 17.1 | 11.7 | 6.84x10^-3 |
| Job category | professional | 148 | 34.1 | 460 | 35.3 | 0.0153 |
|  | intermediate | 119 | 27.4 | 342 | 26.3 |  |
|  | routine | 167 | 38.5 | 500 | 38.4 |  |
| Location | North East | 11 | 2.5 | 44 | 3.4 | 6.32x10^-3 |
|  | North West | 51 | 11.8 | 130 | 10 |  |
|  | Yorkshire | 42 | 9.7 | 132 | 10.1 |  |
|  | East Midlands | 41 | 9.4 | 110 | 8.4 |  |
|  | West Midlands | 27 | 6.2 | 102 | 7.8 |  |
|  | East England | 33 | 7.6 | 106 | 8.1 |  |
|  | South East | 57 | 13.1 | 194 | 14.9 |  |
|  | South West | 51 | 11.8 | 131 | 10.1 |  |
|  | London | 33 | 7.6 | 90 | 6.9 |  |
|  | Wales | 32 | 7.4 | 97 | 7.5 |  |
|  | Scotland | 35 | 8.1 | 113 | 8.7 |  |
|  | Northern Ireland | 21 | 4.8 | 52 | 4 |  |
| Household size | mean (sd) | 2.5 | 1.2 | 2.6 | 1.1 | -0.0458 |
| Baseline household income | mean (sd) | 38.5 | 22.6 | 39.5 | 21.7 | -0.046 |
| Number of comorbidities | mean (sd) | 3.6 | 2.6 | 3.2 | 2.2 | 0.139 |
| N |  | 434 |  | 1302 |  |  |
| *Note.* SMD=standardised mean difference | | | | | | |
