# Supplementary material for: Long-term health conditions and UK labour market outcomes during the COVID-19 pandemic
Source: PLoS One. 2024 May 10;19(5):e0302746. doi: 10.1371/journal.pone.0302746 (PMC11086911; doi:10.1371/journal.pone.0302746)
Supplement: S23 Table — (DOCX) [file pone.0302746.s024.docx]

**Table S23. Liver conditions Mahalanobis distance matching for pre-COVID-19 data.**

|  |  | Treatment | | Control | | SMD |
| --- | --- | --- | --- | --- | --- | --- |
|  |  | N | % | N | % |  |
| Age | mean (sd) | 47.7 | 11.7 | 47.2 | 11.5 | 0.0367 |
| Female |  | 252 | 50.8 | 753 | 50.6 | 4.03x10^-3 |
| White |  | 423 | 85.3 | 1271 | 85.4 | -3.79x10^-3 |
| Baseline hours worked | mean (sd) | 35.2 | 17.9 | 35.3 | 16.6 | -3.77x10^-3 |
| Baseline earnings | mean (sd) | 17.5 | 11.7 | 17.6 | 11.2 | -0.0131 |
| Job category | professional | 210 | 42.3 | 639 | 42.9 | 0.0115 |
|  | intermediate | 114 | 23 | 339 | 22.8 |  |
|  | routine | 172 | 34.7 | 510 | 34.3 |  |
| Location | North East | 17 | 3.4 | 36 | 2.4 | 5.32x10^-3 |
|  | North West | 47 | 9.5 | 152 | 10.2 |  |
|  | Yorkshire | 43 | 8.7 | 129 | 8.7 |  |
|  | East Midlands | 38 | 7.7 | 111 | 7.5 |  |
|  | West Midlands | 41 | 8.3 | 139 | 9.3 |  |
|  | East England | 41 | 8.3 | 134 | 9 |  |
|  | South East | 72 | 14.5 | 203 | 13.6 |  |
|  | South West | 42 | 8.5 | 151 | 10.1 |  |
|  | London | 66 | 13.3 | 175 | 11.8 |  |
|  | Wales | 30 | 6 | 100 | 6.7 |  |
|  | Scotland | 38 | 7.7 | 111 | 7.5 |  |
|  | Northern Ireland | 21 | 4.2 | 47 | 3.2 |  |
| Household size | mean (sd) | 2.9 | 1.4 | 2.9 | 1.3 | -1.52x10^-3 |
| Baseline household income | mean (sd) | 43.9 | 24.9 | 42.2 | 21.2 | 0.066 |
| Number of comorbidities | mean (sd) | 3.1 | 2.3 | 2.8 | 2.2 | 0.115 |
| N |  | 496 |  | 1488 |  |  |
| *Note.* SMD=standardised mean difference | | | | | | |
