# Supplementary material for: Long-term health conditions and UK labour market outcomes during the COVID-19 pandemic
Source: PLoS One. 2024 May 10;19(5):e0302746. doi: 10.1371/journal.pone.0302746 (PMC11086911; doi:10.1371/journal.pone.0302746)
Supplement: S24 Table — (DOCX) [file pone.0302746.s025.docx]

**Table S24. Epilepsy Mahalanobis distance matching for COVID-19 pre-COVID-19 data.**

|  |  | Treatment | | Control | | SMD |
| --- | --- | --- | --- | --- | --- | --- |
|  |  | N | % | N | % |  |
| Age | mean (sd) | 42.7 | 11.9 | 42.8 | 12 | -0.014 |
| Female |  | 130 | 53.1 | 390 | 53.1 | 0 |
| White |  | 215 | 87.8 | 646 | 87.9 | -4.15x10^-3 |
| Baseline hours worked | mean (sd) | 36.3 | 19.7 | 36.8 | 18.5 | -0.0251 |
| Baseline earnings | mean (sd) | 16.7 | 12.2 | 17.3 | 11.4 | -0.0438 |
| Job category | professional | 94 | 38.4 | 285 | 38.8 | -1.56x10^-3 |
|  | intermediate | 59 | 24.1 | 170 | 23.1 |  |
|  | routine | 92 | 37.6 | 280 | 38.1 |  |
| Location | North East | 9 | 3.7 | 17 | 2.3 | -8.99x10^-3 |
|  | North West | 21 | 8.6 | 82 | 11.2 |  |
|  | Yorkshire | 21 | 8.6 | 54 | 7.3 |  |
|  | East Midlands | 22 | 9 | 65 | 8.8 |  |
|  | West Midlands | 21 | 8.6 | 54 | 7.3 |  |
|  | East England | 25 | 10.2 | 84 | 11.4 |  |
|  | South East | 28 | 11.4 | 91 | 12.4 |  |
|  | South West | 20 | 8.2 | 74 | 10.1 |  |
|  | London | 31 | 12.7 | 80 | 10.9 |  |
|  | Wales | 17 | 6.9 | 53 | 7.2 |  |
|  | Scotland | 22 | 9 | 55 | 7.5 |  |
|  | Northern Ireland | 8 | 3.3 | 26 | 3.5 |  |
| Household size | mean (sd) | 2.9 | 1.3 | 2.9 | 1.2 | 0.0119 |
| Baseline household income | mean (sd) | 41.2 | 31.7 | 41.3 | 26.9 | -1.84x10^-3 |
| Number of comorbidities | mean (sd) | 2.1 | 2.1 | 1.9 | 2 | 0.0742 |
| N |  | 245 |  | 735 |  |  |
| *Note.* SMD=standardised mean difference | | | | | | |
