# Supplementary material for: Long-term health conditions and UK labour market outcomes during the COVID-19 pandemic
Source: PLoS One. 2024 May 10;19(5):e0302746. doi: 10.1371/journal.pone.0302746 (PMC11086911; doi:10.1371/journal.pone.0302746)
Supplement: S25 Table — (DOCX) [file pone.0302746.s026.docx]

**Table S25. Pre-COVID-19 analysis employment results.**

|  | Asthma | | Arthritis | | Cancer | | Diabetes | | ENP | | Vascular | | Pulmonary | | Liver | | Epilepsy | |
| --- | --- | --- | --- | --- | --- | --- | --- | --- | --- | --- | --- | --- | --- | --- | --- | --- | --- | --- |
|  | Coeff. | *p* | Coeff. | *p* | Coeff. | *p* | Coeff. | *p* | Coeff. | *p* | Coeff. | *p* | Coeff. | *p* | Coeff. | *p* | Coeff. | *p* |
| LTC | 0.232 | 0.449 | 0.192 | 0.668 | -0.328 | 0.716 | -0.799 | >.999 | -0.417 | 0.474 | -0.298 | 0.504 | 0.736 | >.999 | -0.712 | >.999 | 0.566 | >.999 |
| *t* | -1.2 | 0.000* | -1.27 | 0.000* | -1.43 | 0.002* | -1.29 | >.999 | -1.17 | 0.000* | -1.41 | 0.000* | -1.01 | >.999 | -1.03 | >.999 | -1.4 | >.999 |
| LTC × *t* | -0.0851 | 0.667 | -0.0627 | 0.826 | 0.164 | 0.779 | 0.0513 | >.999 | 0.0376 | 0.918 | 0.118 | 0.685 | -0.294 | >.999 | 0.0282 | >.999 | -0.219 | >.999 |
| ln age | 1.11 | 0.000* | 0.446 | 0.091 | -2.14 | 0.020* | -1.01 | >.999 | 1.52 | 0.000* | -0.258 | 0.358 | -1.82 | >.999 | -0.19 | >.999 | 3.3 | >.999 |
| Female | -0.412 | 0.000* | 0.1 | 0.537 | -0.0237 | 0.948 | -0.181 | >.999 | -0.347 | 0.142 | -0.152 | 0.37 | -0.41 | >.999 | -0.208 | >.999 | -2.08 | >.999 |
| White | 0.0161 | 0.918 | 0.0875 | 0.711 | 0.808 | 0.195 | -0.609 | >.999 | 0.202 | 0.514 | 0.185 | 0.42 | -0.126 | >.999 | -0.771 | >.999 | 1.79 | >.999 |
| Household size | 0.0434 | 0.313 | 0.199 | 0.009* | 0.32 | 0.069 | 0.0428 | >.999 | 0.0229 | 0.799 | 0.217 | 0.003* | 0.0279 | >.999 | -0.13 | >.999 | 0.0633 | >.999 |
| Baseline hours worked | 0.0366 | 0.000* | 0.0436 | 0.000* | 0.0145 | 0.11 | 0.0365 | >.999 | 0.0324 | 0.000* | 0.0359 | 0.000* | 0.052 | >.999 | -3.16x10^-3 | >.999 | 0.0395 | >.999 |
| Baseline earnings | 9.30x10^-3 | 0.043* | 0.0352 | 0.000* | 0.048 | 0.023* | 0.043 | >.999 | 0.0179 | 0.223 | 0.0157 | 0.012* | -3.90x10^-3 | >.999 | 0.0914 | >.999 | 6.61x10^-3 | >.999 |
| Baseline household income | -3.55x10^-4 | 0.691 | -5.03x10^-3 | 0.09 | -6.72x10^-3 | 0.155 | -6.71x10^-5 | >.999 | 1.72x10^-5 | 0.996 | -5.84x10^-3 | 0.019* | 4.48x10^-3 | >.999 | -0.0172 | >.999 | -0.0139 | >.999 |
| Location - North East | 0.556 | 0.125 | 1.25 | 0.051 | -0.678 | 0.529 | 323 | >.999 | 0.813 | 0.298 | 0.263 | 0.616 | 99.5 | >.999 | 226 | >.999 | -3.63 | >.999 |
| Location - North West | 0.131 | 0.538 | 0.16 | 0.62 | -0.026 | 0.972 | -0.794 | >.999 | -6.86x10^-3 | 0.985 | 0.0317 | 0.92 | -0.214 | >.999 | 0.412 | >.999 | -1.81 | >.999 |
| Location - Yorkshire | 0.45 | 0.06 | 0.586 | 0.108 | -0.428 | 0.548 | 0.692 | >.999 | 0.404 | 0.351 | 0.542 | 0.147 | 0.706 | >.999 | 0.69 | >.999 | 0.221 | >.999 |
| Location - East Midlands | 0.108 | 0.652 | 0.0785 | 0.819 | -0.412 | 0.572 | -0.219 | >.999 | 0.405 | 0.365 | 0.254 | 0.486 | 1.4 | >.999 | 0.572 | >.999 | -0.251 | >.999 |
| Location - West Midlands | -0.117 | 0.6 | -0.173 | 0.593 | -0.333 | 0.63 | -0.684 | >.999 | 0.17 | 0.703 | -0.224 | 0.489 | -0.232 | >.999 | 0.405 | >.999 | -1.66 | >.999 |
| Location - East England | 0.0511 | 0.819 | 0.253 | 0.474 | 6.26x10^-3 | 0.993 | 0.0277 | >.999 | 0.491 | 0.279 | -0.13 | 0.7 | 0.349 | >.999 | 0.273 | >.999 | 0.557 | >.999 |
| Location - South East | 0.094 | 0.644 | 0.0382 | 0.897 | -0.0722 | 0.909 | -0.169 | >.999 | -0.0162 | 0.963 | 0.275 | 0.381 | 0.0611 | >.999 | 0.58 | >.999 | -0.395 | >.999 |
| Location - South West | -0.137 | 0.529 | 0.0253 | 0.938 | 0.538 | 0.482 | -0.0125 | >.999 | -0.0543 | 0.887 | -0.154 | 0.628 | -0.161 | >.999 | 0.182 | >.999 | -1.84 | >.999 |
| Location - Wales | 0.259 | 0.318 | 0.273 | 0.464 | 1.65 | 0.205 | 0.503 | >.999 | 0.45 | 0.339 | 0.198 | 0.602 | 1.07 | >.999 | 0.253 | >.999 | 524 | >.999 |
| Location - Scotland | 0.15 | 0.521 | 0.196 | 0.561 | -0.165 | 0.829 | 0.159 | >.999 | -0.101 | 0.789 | 0.101 | 0.769 | 0.811 | >.999 | 0.863 | >.999 | 571 | >.999 |
| Location - Northern Ireland | 0.07 | 0.799 | 0.375 | 0.389 | -0.14 | 0.873 | -0.23 | >.999 | 1.03 | 0.155 | -0.202 | 0.611 | 0.0344 | >.999 | 1.23 | >.999 | 358 | >.999 |
| Number of comorbidities | -0.161 | 0.000* | -0.155 | 0.001* | -0.0788 | 0.296 | -0.0784 | >.999 | -0.121 | 0.015* | -0.118 | 0.005* | -0.098 | >.999 | -0.0647 | >.999 | -0.326 | >.999 |
| Constant | 1.92 | 0.006* | 3.58 | 0.004* | 14.4 | 0.001* | 11 | >.999 | -0.34 | 0.789 | 7.44 | 0.000* | 12 | >.999 | 8.02 | >.999 | -4.41 | >.999 |
| N respondents | 18020 |  | 8444 |  | 2066 |  | 2654 |  | 4542 |  | 8852 |  | 1736 |  | 1984 |  | 980 |  |
| N observations | 47579 |  | 23188 |  | 5700 |  | 7213 |  | 12334 |  | 24055 |  | 4839 |  | 5413 |  | 2606 |  |
| *Note.* LTC=Long-term condition; *t*=0,1,2 signifies Understanding Society main survey waves 7, 8, 9; ENP=emotional, nervous, or psychiatric problem; Coeff.=coefficient; *=significant at 5% level | | | | | | | | | | | | | | | | | | |
