# Supplementary material for: Long-term health conditions and UK labour market outcomes during the COVID-19 pandemic
Source: PLoS One. 2024 May 10;19(5):e0302746. doi: 10.1371/journal.pone.0302746 (PMC11086911; doi:10.1371/journal.pone.0302746)
Supplement: S26 Table — (DOCX) [file pone.0302746.s027.docx]

**Table S26. Pre-COVID-19 analysis hours worked conditional on employment results.**

|  | Asthma | | Arthritis | | Cancer | | Diabetes | | ENP | | Vascular | | Pulmonary | | Liver | | Epilepsy | |
| --- | --- | --- | --- | --- | --- | --- | --- | --- | --- | --- | --- | --- | --- | --- | --- | --- | --- | --- |
|  | Coeff. | *p* | Coeff. | *p* | Coeff. | *p* | Coeff. | *p* | Coeff. | *p* | Coeff. | *p* | Coeff. | *p* | Coeff. | *p* | Coeff. | *p* |
| LTC | 0.194 | 0.535 | 0.0416 | 0.93 | 0.352 | 0.672 | -0.0854 | 0.925 | -0.253 | 0.713 | 0.087 | 0.846 | -0.0712 | 0.958 | -0.379 | 0.739 | -0.22 | 0.894 |
| *t* | 0.275 | 0.032* | 0.318 | 0.095 | -0.492 | 0.162 | 0.349 | 0.378 | 0.803 | 0.002* | -0.0141 | 0.94 | 0.226 | 0.544 | 0.65 | 0.041* | 0.516 | 0.269 |
| LTC × *t* | 0.048 | 0.782 | 7.59x10^-3 | 0.977 | -0.0444 | 0.925 | -0.268 | 0.597 | -0.244 | 0.516 | 0.179 | 0.471 | -0.0834 | 0.906 | -0.391 | 0.534 | 0.273 | 0.755 |
| ln age | -2.24 | 0.000* | -2.88 | 0.000* | -4.37 | 0.000* | -2.87 | 0.001* | -2.25 | 0.000* | -2.78 | 0.000* | -1.91 | 0.107 | -1.86 | 0.014* | -1.53 | 0.2 |
| Female | -1.52 | 0.000* | -1.21 | 0.000* | -1.17 | 0.002* | -1.46 | 0.000* | -1.52 | 0.000* | -1.31 | 0.000* | -0.42 | 0.375 | -0.773 | 0.057 | -2.35 | 0.000* |
| White | 0.388 | 0.027* | 0.225 | 0.428 | 0.9 | 0.22 | 0.585 | 0.228 | 0.503 | 0.204 | 0.588 | 0.023* | -0.143 | 0.905 | 0.667 | 0.269 | 2.4 | 0.049* |
| Household size | -0.0651 | 0.116 | 5.76x10^-3 | 0.932 | 0.191 | 0.188 | -0.218 | 0.074 | -0.126 | 0.206 | 0.0892 | 0.172 | 0.313 | 0.145 | 0.371 | 0.027* | -0.382 | 0.135 |
| Baseline hours worked | 0.726 | 0.000* | 0.74 | 0.000* | 0.681 | 0.000* | 0.762 | 0.000* | 0.717 | 0.000* | 0.743 | 0.000* | 0.788 | 0.000* | 0.734 | 0.000* | 0.716 | 0.000* |
| Baseline earnings | 0.0707 | 0.000* | 0.108 | 0.000* | 0.147 | 0.000* | 0.0727 | 0.000* | 0.0867 | 0.000* | 0.0736 | 0.000* | 0.0945 | 0.000* | 0.126 | 0.000* | 0.086 | 0.005* |
| Baseline household income | -5.69x10^-4 | 0.322 | -6.38x10^-3 | 0.038* | -0.0243 | 0.000* | -2.68x10^-4 | 0.693 | -9.71x10^-6 | 0.991 | -9.97x10^-3 | 0.000* | -0.047 | 0.000* | -0.0327 | 0.004* | -0.0186 | 0.07 |
| Location - North East | 0.331 | 0.364 | 0.571 | 0.267 | 0.369 | 0.848 | 1.37 | 0.102 | 0.792 | 0.238 | 0.195 | 0.685 | 1.72 | 0.304 | 1.55 | 0.102 | 2.58 | 0.117 |
| Location - North West | -0.499 | 0.046* | -0.241 | 0.536 | 0.0343 | 0.968 | -1.26 | 0.092 | -0.556 | 0.353 | -0.626 | 0.074 | 1.42 | 0.258 | -1.83 | 0.034* | -1.47 | 0.328 |
| Location - Yorkshire | -0.282 | 0.283 | -0.415 | 0.346 | -0.868 | 0.387 | -1.03 | 0.18 | -0.693 | 0.253 | -0.939 | 0.017* | 0.07 | 0.957 | -1.87 | 0.026* | -1.87 | 0.203 |
| Location - East Midlands | -0.249 | 0.374 | -0.275 | 0.528 | -0.953 | 0.328 | -1.55 | 0.059 | 0.497 | 0.411 | -0.764 | 0.048* | 0.797 | 0.546 | -1.84 | 0.024* | -1.44 | 0.336 |
| Location - West Midlands | -0.0877 | 0.708 | 0.148 | 0.683 | -2 | 0.019* | -0.0853 | 0.871 | 0.2 | 0.679 | -0.274 | 0.409 | 2.02 | 0.095 | -0.772 | 0.297 | -1.08 | 0.44 |
| Location - East England | -0.0568 | 0.821 | -0.137 | 0.732 | -0.577 | 0.543 | -1.25 | 0.062 | 0.0139 | 0.98 | -0.467 | 0.215 | -0.112 | 0.933 | -1.67 | 0.038* | -0.772 | 0.578 |
| Location - South East | -0.459 | 0.05 | -0.533 | 0.153 | -0.652 | 0.423 | -1.37 | 0.051 | -0.605 | 0.255 | -0.517 | 0.125 | 0.824 | 0.502 | -1.17 | 0.115 | -1.56 | 0.254 |
| Location - South West | -0.111 | 0.672 | 0.133 | 0.739 | 0.636 | 0.446 | -1.67 | 0.029* | -0.214 | 0.696 | -0.349 | 0.342 | 0.905 | 0.471 | -0.948 | 0.221 | -1.54 | 0.265 |
| Location - Wales | -0.0472 | 0.863 | -0.0522 | 0.907 | -0.203 | 0.831 | -1.44 | 0.034* | -0.545 | 0.386 | -0.452 | 0.259 | 0.392 | 0.786 | -1.66 | 0.036* | -1.92 | 0.228 |
| Location - Scotland | -0.193 | 0.452 | 0.115 | 0.769 | -1.05 | 0.257 | -0.694 | 0.303 | -0.0937 | 0.854 | -0.48 | 0.199 | 1.99 | 0.115 | -0.53 | 0.474 | -1.71 | 0.345 |
| Location - Northern Ireland | -0.424 | 0.191 | -0.49 | 0.356 | -0.0291 | 0.977 | -1.46 | 0.163 | -0.662 | 0.39 | -0.109 | 0.817 | -0.513 | 0.79 | -2.01 | 0.235 | -2.85 | 0.113 |
| Number of comorbidities | -0.0249 | 0.566 | -0.0249 | 0.656 | 0.118 | 0.184 | 0.0999 | 0.246 | 0.0483 | 0.472 | 7.92x10^-3 | 0.871 | -0.0889 | 0.367 | 0.148 | 0.138 | 0.056 | 0.73 |
| Constant | 17.7 | 0.000* | 19.4 | 0.000* | 26.5 | 0.000* | 20 | 0.000* | 17.7 | 0.000* | 19.5 | 0.000* | 14.5 | 0.004* | 15.4 | 0.000* | 15.7 | 0.001* |
| N respondents | 18020 |  | 8444 |  | 2066 |  | 2654 |  | 4542 |  | 8852 |  | 1736 |  | 1984 |  | 980 |  |
| N observations | 47579 |  | 23188 |  | 5700 |  | 7213 |  | 12334 |  | 24055 |  | 4839 |  | 5413 |  | 2606 |  |
| *Note.* LTC=Long-term condition; *t*=0,1,2 signifies Understanding Society main survey waves 7, 8, 9; ENP=emotional, nervous, or psychiatric problem; Coeff.=coefficient; *=significant at 5% level | | | | | | | | | | | | | | | | | | |
