# Supplementary material for: Long-term health conditions and UK labour market outcomes during the COVID-19 pandemic
Source: PLoS One. 2024 May 10;19(5):e0302746. doi: 10.1371/journal.pone.0302746 (PMC11086911; doi:10.1371/journal.pone.0302746)
Supplement: S27 Table — (DOCX) [file pone.0302746.s028.docx]

**Table S27. Pre-COVID-19 analysis earnings conditional on employment results.**

|  | Asthma | | Arthritis | | Cancer | | Diabetes | | ENP | | Vascular | | Pulmonary | | Liver | | Epilepsy | |
| --- | --- | --- | --- | --- | --- | --- | --- | --- | --- | --- | --- | --- | --- | --- | --- | --- | --- | --- |
|  | Coeff. | *p* | Coeff. | *p* | Coeff. | *p* | Coeff. | *p* | Coeff. | *p* | Coeff. | *p* | Coeff. | *p* | Coeff. | *p* | Coeff. | *p* |
| LTC | 0.0412 | 0.834 | -0.0285 | 0.914 | 0.17 | 0.798 | -0.0225 | 0.966 | -4.26x10^-3 | 0.991 | 0.0338 | >.999 | 0.0269 | 0.967 | -0.28 | 0.717 | 0.0558 | 0.952 |
| *t* | 0.58 | 0.000* | 0.487 | 0.000* | 0.165 | 0.526 | 0.516 | 0.018* | 0.715 | 0.000* | 0.48 | >.999 | 0.409 | 0.029* | 0.529 | 0.008* | 0.647 | 0.027* |
| LTC × *t* | -0.195 | 0.061 | -0.16 | 0.26 | 0.0972 | 0.788 | -0.446 | 0.12 | -0.0812 | 0.691 | -0.111 | >.999 | -0.129 | 0.717 | 0.012 | 0.977 | 0.014 | 0.979 |
| ln age | -0.273 | 0.055 | -0.682 | 0.002* | -1.44 | 0.053 | -0.893 | 0.103 | 0.0679 | 0.793 | -0.878 | >.999 | -0.563 | 0.351 | -1.05 | 0.026* | 0.474 | 0.491 |
| Female | -1.14 | 0.000* | -0.949 | 0.000* | -1.23 | 0.000* | -1.13 | 0.000* | -1.13 | 0.000* | -1.01 | >.999 | -0.596 | 0.010* | -0.545 | 0.022* | -1.04 | 0.001* |
| White | 0.263 | 0.014* | 0.119 | 0.439 | 0.0548 | 0.903 | -0.0673 | 0.818 | 0.0586 | 0.808 | 0.483 | >.999 | -0.818 | 0.075 | 0.148 | 0.644 | -0.614 | 0.244 |
| Household size | 0.0151 | 0.568 | 0.0845 | 0.023* | 0.0788 | 0.461 | -0.0567 | 0.46 | 0.0279 | 0.586 | 0.0786 | >.999 | 0.0451 | 0.654 | 0.116 | 0.171 | -0.091 | 0.503 |
| Baseline hours worked | 0.0264 | 0.000* | 0.0288 | 0.000* | 0.0382 | 0.000* | 0.0614 | 0.000* | 0.014 | 0.000* | 0.03 | >.999 | 0.0415 | 0.000* | 0.0254 | 0.000* | 0.0141 | 0.087 |
| Baseline earnings | 0.799 | 0.000* | 0.82 | 0.000* | 0.78 | 0.000* | 0.758 | 0.000* | 0.836 | 0.000* | 0.808 | >.999 | 0.837 | 0.000* | 0.863 | 0.000* | 0.84 | 0.000* |
| Baseline household income | -8.66x10^-4 | 0.000* | -7.02x10^-3 | 0.000* | 4.50x10^-4 | 0.874 | 3.01x10^-4 | 0.546 | 1.81x10^-4 | 0.78 | -7.73x10^-3 | >.999 | -0.0128 | 0.006* | -8.45x10^-3 | 0.065 | -2.58x10^-3 | 0.528 |
| Location - North East | -0.718 | 0.002* | -0.854 | 0.013* | -1.84 | 0.132 | -0.955 | 0.388 | -0.8 | 0.091 | -1.08 | >.999 | -0.105 | 0.883 | -0.831 | 0.363 | -2.08 | 0.032* |
| Location - North West | -1.03 | 0.000* | -1.03 | 0.000* | -1.98 | 0.000* | -1.15 | 0.004* | -1.18 | 0.000* | -0.87 | >.999 | 0.323 | 0.452 | -1.13 | 0.013* | -1.88 | 0.018* |
| Location - Yorkshire | -0.886 | 0.000* | -0.9 | 0.000* | -1.43 | 0.027* | -0.911 | 0.16 | -1.14 | 0.003* | -1.31 | >.999 | 0.127 | 0.829 | -1.09 | 0.030* | -1.59 | 0.002* |
| Location - East Midlands | -0.959 | 0.000* | -0.8 | 0.000* | -1.1 | 0.052 | -1.26 | 0.001* | -0.723 | 0.015* | -1.09 | >.999 | 0.127 | 0.775 | -1.07 | 0.006* | -1.34 | 0.059 |
| Location - West Midlands | -0.973 | 0.000* | -0.994 | 0.000* | -2.73 | 0.000* | -1.08 | 0.008* | -1.24 | 0.000* | -1.15 | >.999 | 0.0832 | 0.855 | -1.12 | 0.006* | -2.87 | 0.000* |
| Location - East England | -0.581 | 0.000* | -0.558 | 0.003* | -1.28 | 0.008* | -0.572 | 0.117 | -0.512 | 0.026* | -0.969 | >.999 | 0.241 | 0.607 | -1.94 | 0.000* | -0.945 | 0.142 |
| Location - South East | -0.515 | 0.000* | -0.569 | 0.002* | -0.662 | 0.167 | -0.458 | 0.253 | -0.677 | 0.006* | -0.641 | >.999 | 0.307 | 0.475 | -0.512 | 0.176 | -1.98 | 0.003* |
| Location - South West | -0.802 | 0.000* | -0.741 | 0.000* | -0.756 | 0.09 | -0.489 | 0.169 | -0.867 | 0.000* | -0.902 | >.999 | 0.548 | 0.204 | -0.872 | 0.022* | -1.76 | 0.002* |
| Location - Wales | -0.945 | 0.000* | -0.95 | 0.000* | -1.5 | 0.073 | -1.22 | 0.014* | -1.27 | 0.000* | -1.16 | >.999 | 0.189 | 0.745 | -1.39 | 0.011* | -1.97 | 0.012* |
| Location - Scotland | -0.711 | 0.000* | -0.557 | 0.006* | -1.06 | 0.081 | -0.642 | 0.336 | -0.68 | 0.008* | -0.94 | >.999 | 0.552 | 0.243 | -0.429 | 0.358 | -2.06 | 0.005* |
| Location - Northern Ireland | -0.992 | 0.000* | -0.71 | 0.014* | -1.32 | 0.089 | -0.831 | 0.222 | -0.847 | 0.061 | -0.819 | >.999 | 0.423 | 0.52 | -0.075 | 0.9 | -1.32 | 0.124 |
| Number of comorbidities | -0.0848 | 0.002* | -0.0521 | 0.06 | -0.0285 | 0.64 | -5.58x10^-3 | 0.925 | -0.0994 | 0.004* | -0.0838 | >.999 | -0.0746 | 0.139 | 0.0173 | 0.735 | -0.156 | 0.1 |
| Constant | 4.08 | 0.000* | 5.39 | 0.000* | 8.91 | 0.005* | 6.25 | 0.007* | 2.94 | 0.006* | 6.24 | >.999 | 4.64 | 0.064 | 6.16 | 0.001* | 3.1 | 0.25 |
| N respondents | 18020 |  | 8444 |  | 2066 |  | 2654 |  | 4542 |  | 8852 |  | 1736 |  | 1984 |  | 980 |  |
| N observations | 47579 |  | 23188 |  | 5700 |  | 7213 |  | 12334 |  | 24055 |  | 4839 |  | 5413 |  | 2606 |  |
| *Note.* LTC=Long-term condition; *t*=0,1,2 signifies Understanding Society main survey waves 7, 8, 9; ENP=emotional, nervous, or psychiatric problem; Coeff.=coefficient; *=significant at 5% level | | | | | | | | | | | | | | | | | | |
